# Facile Assembling of Novel 2,3,6,7,9-pentaazabicyclo- [3.3.1]nona-3,7-diene Derivatives under Microwave and Ultrasound Platforms

**DOI:** 10.3390/molecules24061110

**Published:** 2019-03-20

**Authors:** Hamad M. Al-Matar, Kamal M. Dawood, Wael M. Tohamy, Mona A. Shalaby

**Affiliations:** 1 Chemistry Department, Faculty of Science, University of Kuwait, P.O. Box 5969, Safat 13060, Kuwait; monashalby203@gmail.com; 2 Department of Chemistry, Faculty of Science, Cairo University, Giza 12613, Egypt; 3 Organometallic and Organometalloid Chemistry Department, National Research Centre, Cairo 12622, Egypt; wael_tohamy79@hotmail.com

**Keywords:** Bicyclo[3.3.1]nonadienes, 3-oxo-2-arylhydrazono-propanals, catalysis, ultrasound, microwave

## Abstract

Reactions of a series of 3-oxo-2-arylhydrazonopropanal derivatives with two molar ratio of ammonium acetate afforded a library of tetrasubstituted 2,3,6,7,9-pentaazabicyclo[3.3.1]nona- 3,7-diene derivatives in good to excellent isolated yields. The reaction was activated with triethylamine catalyst under three different heating modes: thermal, ultrasonic and microwave irradiating conditions in ethanol solvent. The structures of the isolated products were fully characterized by spectral and analytical data as well as X-ray single crystal of selected examples.

## 1. Introduction

**Figure 1:**
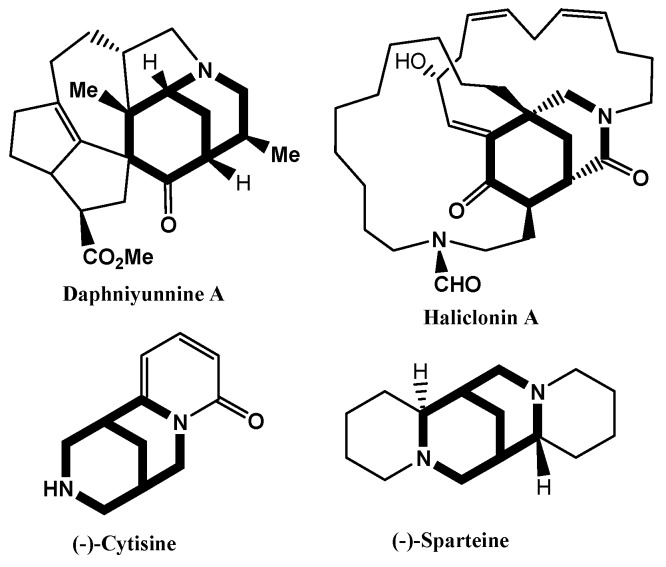
Azabicyclo[3.3.1]nonane-based natural products.

The azabicyclo[3.3.1]nonane moiety is a privileged scaffold embedded in the structures of numerous bioactive natural products (Figure 1) [1,2]. The azabicyclo[3.3.1]nonane derivatives are reported to have diverse biological applications. For example, 1-azabicyclo[3.3.1]nonanes are useful for the treatment of psychotic and neurodegenerative disorders [3,4]. The 2-azabicyclo[3.3.1]nonane skeleton is present in several important narcotic analgesics and marine alkaloids [5,6,7]. 3-Azabicylo[3.3.1]nonane is the core substructure of the marine natural product; *Haliclonin A* (Figure 1) [8,9]. 9-Azabicyclo[3.3.1]nonane derivatives possess cytotoxic [10], dopamine D3 receptor ligands [11], high sigma-2 receptor affinities [12], and are used for the treatment of diabetes mellitus [13]. Furthermore, 1,4-diazabicyclo[3.3.1]nonane derivatives are reported to exhibit high in vivo affinity and selectivity for the dopamine transporter (DAT) blockers [14,15]. 3,7-Diazabicyclo[3.3.1]nonanes are reported to be useful in the treatment of cardiac arrhythmias [16], and exhibited anti-platelet, antithrombotic activities [17], as well as high affinities at various nicotinic acetylcholine receptors (nAChRs) [18,19,20]. 3,9-Diazabicyclo[3.3.1]nonanes showed the 5-HT3 receptor antagonist [21] and opioid δ and μ-receptor activities [22,23]. Triazabicyclo[3.3.1]nonane derivatives such as 2,6,9- and 3,7,9-triazabicyclo[3.3.1]nonanes [24,25,26,27,28] were synthesized from dimerization of α,β-unsaturated carbonyl compounds with alkylamines. Some 1,3,5,7-tetraazabicyclo[3.3.1]nonane derivatives have antithrombotic activities [29]. Although tremendous progress has been achieved in the synthesis of mono-, di-, and tri-azabicyclo[3.3.1]nonanes [1,2,3,4,5,6,7,8,9,10,11,12,13,14,15,16,17,18,19,20,21,22,23,24,25,26,27], the synthesis of tetra- and penta-azabicyclo[3.3.1]nonane frameworks has rarely been disclosed in the literature [30,31,32,33].

The microwave irradiation methodology is widely employed in organic reactions because it has several advantages over conventional heating, resulting in high yields, low by-products, rapid heating and easy purification [34,35,36,37,38,39,40,41]. Mechanistically, microwave irradiation affects the reaction through internal heating by direct coupling of microwave energy with the bulk reaction mixture. Furthermore, the eco-friendly ultrasound platform has receiving much interest due to its high impacts on organic synthesis, medicinal chemistry and materials science [42,43,44,45,46]. It leads to the formation of pure products in high yields and selectivity in a shorter reaction time. In continuation of our research work employing ultrasound and microwave irradiations in the synthesis of biologically active heterocycles [47,48,49,50,51,52,53,54,55,56,57], we envisaged herein an efficient and versatile one-pot protocol for rapid assembly of novel *C_2_*-symmetric 2,3,6,7,9-pentaazabicyclo[3.3.1]nonane derivatives through dimerization of 3-oxo-2-arylhydrazonopropanals with ammonium acetate via a double Mannich-type reaction under three different heating platforms: conventional, ultrasound and microwave irradiation. The structures of the obtained products are established from their single crystal X-ray analysis and spectral data (IR, MS, HRMS, ^1^H- and ^13^C-NMR).

## 2. Results

The intermolecular Mannich reaction is considered to be a powerful route for the synthesis of azacyclic products from acyclic substrates [58]. At first, the investigations focused on screening various reaction parameters (e.g.*,* solvents, bases and heating techniques) for optimizing the reaction conditions of the double-Mannish reaction of the 3-oxo-2-arylhydrazonopropanal derivative **1a** with ammonium acetate were thoroughly evaluated and the reaction was followed by TLC till almost full conversion of the starting substrates and the results are depicted in Table 1. Heating the 3-oxopropanal derivative **1a** with double equivalents of ammonium acetate in ethanol under either reflux temperature (15 h), ultrasound irradiation (US) (120 min at 80 °C and 110 W), or microwave irradiation (MW) (30 min at 80 °C and 200 W) in the absence of catalysts, only a trace amount of product was detected by TLC (run 1, Table 1). When the reaction was heated at reflux using triethylamine (Et_3_N) as catalyst (15 mol%) for 4 h (as examined by TLC), it led to the formation of the 2,3,6,7,9-pentaazabicyclo[3.3.1]nonane derivative **2a** in 70% isolated yield (run 2, Table 1). The structure of reaction product **2a** was confirmed from its spectral data (IR, MS, HRMS, ^1^H- and ^13^C-NMR). ^1^H-NMR spectrum of compound **2a** showed triplet at δ 4.30 (D_2_O-exchangeable) assigned to the NH proton and a doublet at δ 6.76 due to the symmetric-bridgehead H1 and H5-protons in addition to a multiplet at δ 7.3–7.95, corresponding to 16 aromatic protons. ^13^C-NMR spectrum of **2a** exhibited symmetric 11 signals at δ 56.48, 118.94, 127.70, 127.98, 128.92, 129.99, 132.15, 136.98, 138.37, 141.88 and 189.39, corresponding to 30 aromatic and aliphatic carbons. When the same reaction was repeated under US (for 60 min) and MW (for 5 min), the product **2a** was obtained in 82% and 87% yields, respectively (run 2, Table 1). For the same reaction, use of 25 mol% of Et_3_N resulted in a significant increase in the product yield, to 78%, 89% and 94% when the reaction was carried out at reflux (3 h), US (50 min) and MW (3 min), respectively (run 3, Table 1). Further increase in the amount of Et_3_N (30 mol%) could not significantly improve the yield, as shown in run 4, Table 1. Further evaluation of the effect of the molar ratio of **1a** and ammonium acetate in the presence of 25 mol% of Et_3_N was attempted, where product **2a** was formed in 66%, 73% and 80% yields after heating at reflux (3 h), US (50 min) and MW (3 min), respectively, when **1a** and ammonium acetate (1:1 molar ratio) were employed (run 5, Table 1). Repeating the reaction of **1a** and ammonium acetate (2:1 molar ratio) gave 60%, 68% and 73% yields of **2a** upon heating at reflux (3 h), US (50 min) and MW (3 min), respectively (run 6, Table 1). Using methanol or isopropanol solvents instead of ethanol in the presence of Et_3_N (25 mol%) and 1:2 molar ratio of **1a** and ammonium acetate had little effect on the product yields under all heating modes, as shown in runs 7 and 8, Table 1. Non-alcoholic solvents lowered the reaction yields and increased the reaction time, where employing *n*-hexane, acetic acid, dimethylformamide (DMF) or toluene as reaction solvents resulted in the formation of **2a** in 30~35%, 40~52% and 50~65% yields, under reflux, US and MW conditions, respectively, as shown in runs 9–12, Table 1. Keeping ethanol as solvent, further attempts to evaluate the effect of base-types (pyridine, DABCO, DBU, NaHCO_3,_ K_2_CO_3,_ NaOH) on the reaction yields were studied and in all cases, regardless of whether an organic or inorganic base catalyst was employed (25 mol%), the overall yields decreased sharply; 10~20%, 10~28% and 15~35% yields, under the applied activation modes—thermal, US and MW—respectively (runs 13–18, Table 1). From the obtained data in Table 1, it can be concluded that EtOH/Et_3_N is the most effective reaction condition for achieving the stated goals.

**Figure 2:**
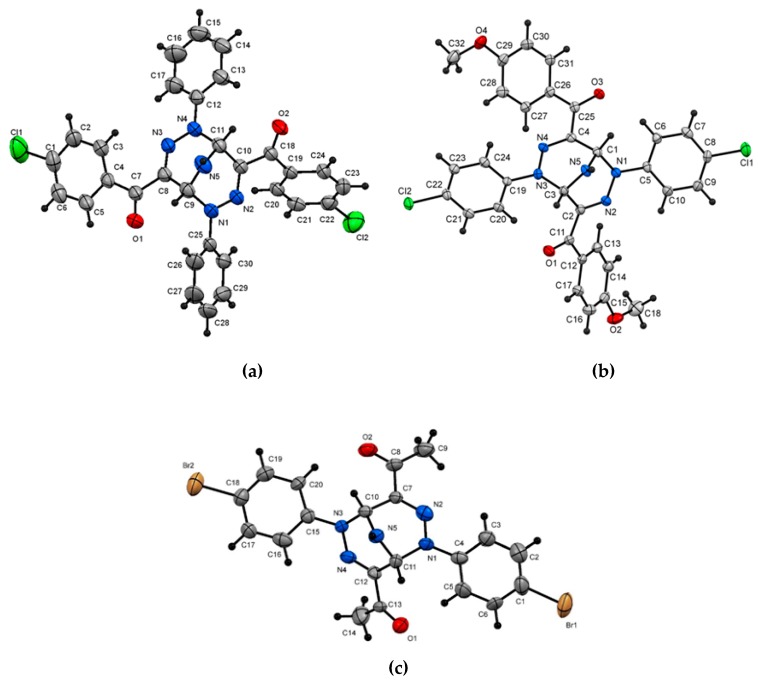
ORTEP diagrams of the crystal structures of **2f** (**a**)**, 2k** (**b**)**, 2p** (**c**)**.**

Next, a variety of C*_2_*-symmetric tetrasubstituted 2,3,6,7,9-pentaazabicyclo[3.3.1]nona-3,7-diene derivatives were prepared in accordance with the above optimized reaction conditions. The use of several 3-oxo-2-arylhydrazonopropanal derivatives **1a**–**p** with ammonium acetate afforded the corresponding double Mannich-type products **2a**–**p** in good to excellent isolated yields and the reactions were followed by TLC until full conversion of the starting substrates (Table 2). It was gratifying to see that the reaction proceeds well, with high isolated yields (81~94%) for all derivatives under microwave irradiation within 3~9 min at 80 °C (200 W). Reaction yields were slightly decreased by conducting the reaction under ultrasound at 80 °C (110 W), with the products being obtained in 73~89% isolated yields. When reactions were carried out under conventional heating mode, the isolated yields varied from 62 to 86% after 3~8 hours when followed by TLC. All the resulted products were established based on their elemental analyses and spectral data (IR, MS, HRMS, ^1^H- and ^13^C-NMR), as well as single crystal X-ray crystallography, of three examples, **2f, 2k** and **2p**, as shown in Figure 2a–c [59]. It is worth mentioning that the bicyclic scaffolds adopt a unique C*_2_*-symmetric V-shaped structures [60].

**Scheme 1:**
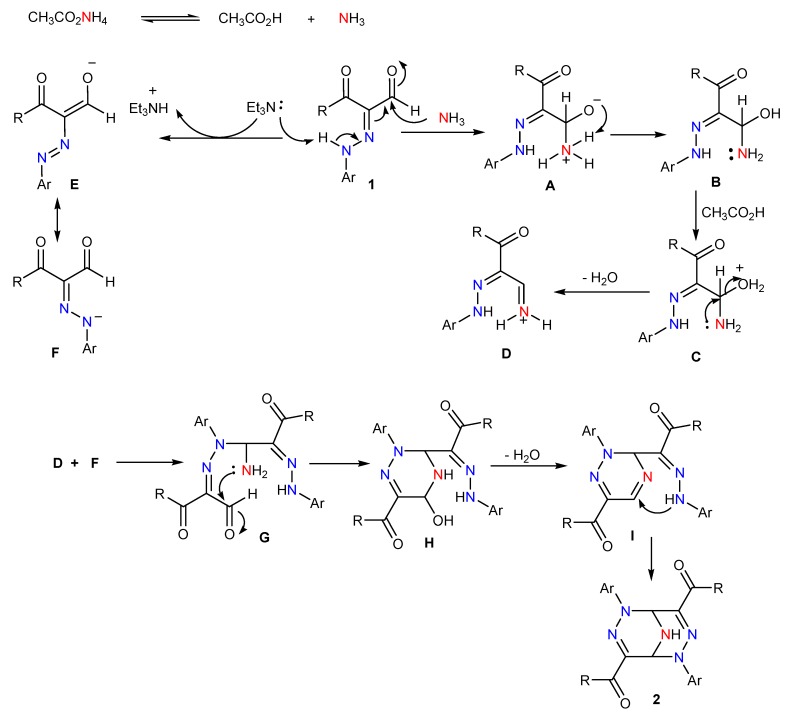
Suggested reaction mechanism for 2,3,6,7,9-pentaazabicyclo[3.3.1]nonanes **2.**

A plausible mechanism is proposed, as outlined in Scheme 1, on the basis of the aforementioned results for the tandem formation of the 2,3,6,7,9-pentaazabicyclo[3.3.1]nonane derivatives **2a**–**p** through a Mannich-type reaction. At first, ammonium acetate is dissociated into ammonia and acetic acid. Then, nucleophilic addition of ammonia to the aldehydic carbonyl of compound **1**, followed by dehydration through the intermediary **A~C** furnished the iminium ion intermediate **D**. A second molecule of structure **1** is deprotonated by Et_3_N as basic catalyst to form the arylazo-enolate ion intermediate **E**. The resonated intermediate **F** attacks the iminium ion intermediate **D** to form the non-isolated Mannich adduct **G** that cyclizes to form the triazacyclic intermediate **H** by intramolecular attacking of the amine function to the aldehyde function. Finally, loss of water molecule from **H** produced the pentaazabicyclo[3.3.1]nonane system **2**.

## 3. Materials and Methods

### 3.1. General Information 

Melting points were recorded on a Griffin melting point apparatus and are reported uncorrected. IR spectra were recorded using KBr disks using a Perkin-Elmer System 2000 FT-IR spectrophotometer (Perkin Elmer, Shelton, CT, USA). ^1^H-NMR (600 MHz) and ^13^C-NMR (150 MHz) spectra were recorded at 25 °C using DMSO-*d*_6_ as solvent with TMS as internal standard on a Bruker DPX 600 super-conducting NMR spectrometer (Bruker, Karlsruhe, Germany). Chemical shifts δ are reported in ppm. Low-resolution electron impact mass spectra [MS (EI)] and high-resolution electron impact mass spectra [HRMS (EI)] were performed using a high-resolution GC-MS (DFS) thermo spectrometer at 70.1 eV using a magnetic sector mass analyzer (Thermo, Bremen, Germany). Follow-up of the reactions and checking homogeneity of the prepared compounds was carried out by using thin layer chromatography (TLC). Microwave experiments were carried out using a CEM Discover Labmate microwave apparatus (300 W with CHEMDRIVER software; Matthews, NC, USA). Reactions were conducted under microwave irradiation in heavy-walled Pyrextubes fitted with PCS caps (closed vessel under pressure). The X-ray crystal structures were determined by using a Rigaku R-AXISRAPID diffractometer (Rigaku, Tokyo, Japan) and Bruker X8 Prospector and the collection of single crystal data was made at room temperature by using Cu-Kα radiation. The data were collected at room temperature. The structures were solved by using direct methods and expanded using Fourier techniques. The non-hydrogen atoms were refined anisotropically. The structures were solved and refined using the Bruker SHELXTLSoftware Package (Structure solution program-SHELXS-97 and Refinement program-SHELXL97) [61].

Data were corrected for the absorption effects using the multi-scan method (SADABS). Sonication was performed in MKC6, Guyson ultrasonic bath (Model-MKC6, operating frequency 38 kHz ± 10% and an output power of 110 Watts) with a digital timer (6 s to 100 min) and a heater, allowing solution heating to be set from 20 to 80 °C in 1 °C increments. The inside tank dimensions are 150 × 300 × 150 mm (length × width × depth) with a fluid capacity of 6 L. The 3-oxo-2-arylhydrazonopropanal derivatives **1a**–**p** were prepared following reported procedures in the literature [62].

### 3.2. Synthesis of 2,3,6,7,9-Pentaazabicyclo[3.3.1]nona-3,7-diene Derivatives ***2a**–**p***

#### 3.2.1. General Method A 

A mixture of the appropriate arylhydrazonopropanals **1a**–**p** (5 mmol) and ammonium acetate (10 mmol) was dissolved in of ethanol (7 mL), then triethylamine (25 mol%) was added and the reaction mixture was refluxed for 3–8 h (monitored by TLC using a mixture of petroleum ether (bp 60–80):EtOAc (2:1)). The reaction mixture was then evaporated under reduced pressure and the solid product, so formed, was recrystallized from EtOH/DMF to give the corresponding 2,3,6,7,9-pentaazabicyclo[3.3.1]nonane products **2a**–**p**.

#### 3.2.2. General Method B

In a round-bottomed three-necked flask, a mixture of the appropriate arylhydrazonopropanals **1a**–**p** (5 mmol) and ammonium acetate (10 mmol) in of ethanol (7 mL), then triethylamine (25 mol%) was added and the reaction mixture was sonicated in a MKC6, Guyson ultrasonic bath (Model-MKC6, operating frequency 38 kHz ± 10% and an output power of 110 W) for 50–110 min at 80 °C. The reaction was controlled by TLC and continued until the starting substrates were completely consumed, then left to cool to room temperature. In each case, the solid product, so formed, was collected by filtration, washed with ethanol, dried and recrystallized from EtOH/DMF to give the corresponding products **2a**–**p**.

#### 3.2.3. General Method C 

In a process glass vial, a mixture of the appropriate arylhydrazonopropanals **1a–p** (5 mmol) and ammonium acetate (10 mmol) in ethanol (7 mL), then triethylamine (25 mol%) was added. The vial was capped properly, and thereafter, the mixture was heated under microwave irradiating conditions at 80 °C and 300 W for the appropriate reaction time as listed in Table 2. After cooling to room temperature, the products were isolated by filtration, washed with ethanol, dried and recrystallized from EtOH/DMF to give the corresponding products **2a**–**p**, see Supplementary materials.

*2,6-Di(4-chlorophenyl)-4,8-di(4-fluorobenzoyl)-2,3,6,7,9-pentaazabicyclo[3.3.1]nona-3,7-diene* (**2a**): Pale yellow color; m.p. 245–246 °C; IR (KBr): 3331, 3073, 1636, 1597 cm^−1^; ^1^H-NMR (DMSO-*d*_6_): δ = 4.30 (t, 1H, *J* = 2.4 Hz, NH), 6.76 (d, 2H, *J* = 2.4 Hz, H-1 and H-5), 7.31 (t, 4H, *J* = 9 Hz), 7.45 (d, 4H, *J* = 9.6 Hz), 7.65 (d, 4H, *J* = 9 Hz), 7.93–7.95 (m, 4H); ^13^C-NMR (DMSO-*d*_6_): δ = 56.93, 115.45, 115.60, 119.49, 128.25, 129.44, 133.39, 133.45, 133.92, 138.74, 142.35, 164.03, 165.69, 188.35; MS (EI, 70 eV): *m/z* (%) = 588.90 (M^+^, 6), 463.0 (34), 314.0 (18), 138.0 (5), 123.0 (100), 95 (29); HRMS (EI): *m/z* calcd for C_30_H_19_Cl_2_F_2_N_5_O_2_: 589.0884; found: 589.0878.

*2,6-Di(4-bromophenyl)-4,8-di(4-fluorobenzoyl)-2,3,6,7,9-pentaazabicyclo[3.3.1]nona-3,7-diene* (**2b**): Yellow color; m.p. 216–217 °C; IR (KBr): 3332, 3065, 1633, 1597 cm^−1^; ^1^H-NMR (DMSO-*d*_6_): δ = 4.30 (t, 1H, *J* = 2.4 Hz, NH), 6.76 (d, 2H, *J* = 2.4 Hz, H-1 and H-5), 7.29–7.33 (m, 4H), 7.56–7.60 (m, 8H), 7.92–7.95 (m, 4H); ^13^C-NMR (DMSO-*d*_6_): δ = 56.37, 114.97, 115.12, 115.83, 119.36, 131.84, 132.91, 132.97, 133.39, 133.41, 138.28, 142.26, 163.55, 165.21, 187.84; MS (EI, 70 eV): *m/z* (%) = 678.80 (M^+^, 5), 508.9 (19), 498.8 (3), 359.9 (10), 181.9 (3), 154.9 (8), 123.0 (100), 95.0 (27); HRMS (EI): *m/z* calcd for C_30_H_19_Br_2_F_2_N_5_O_2_: 676.9874; found: 676.9865.

*4,8-Di(benzoyl)-2,6-di(4-chlorophenyl)-2,3,6,7,9-pentaazabicyclo[3.3.1]nona-3,7-diene* (**2c**): Pale yellow color; m.p. 223–224 °C; IR (KBr): 3323, 3066, 1648, 1600 cm^−1^; ^1^H-NMR (DMSO-*d*_6_): δ = 4.31 (t, 1H, *J* = 2.4 Hz, NH), 6.76 (d, 2H, *J* = 2.4 Hz, H-1 and H-5), 7.44 (d, 4H, *J* = 1.8 Hz), 7.49 (t, 4H, *J* = 7.8 Hz), 7.59 (t, 2H, *J* = 7.5 Hz), 7.63–7.65 (m, 4H), 7.83 (d, 4H, *J* = 7.2 Hz); ^13^C-NMR (DMSO-*d*_6_): δ = 56.48, 118.94, 127.70, 127.98, 128.92, 129.99, 132.15, 136.98, 138.37, 141.88, 189.39; MS (EI, 70 eV): *m/z* (%) = 553.30 (M^+^, 15.9), 448.2 (4), 427.2 (70), 138.1 (5), 127.0 (10), 105.1 (100), 77.0 (33); HRMS (EI): *m/z* calcd for C_30_H_21_Cl_2_N_5_O_2_: 553.1072; found: 553.1067.

*4,8-Di(benzoyl)-2,6-di(4-bromophenyl)-2,3,6,7,9-pentaazabicyclo[3.3.1]nona-3,7-diene* (**2d**): Pale yellow color; m.p. 245–247 °C; IR (KBr): 3334, 2919, 1634, 1593 cm^−1^; ^1^H-NMR (DMSO-*d*_6_): δ = 4.34 (t, 1H, *J* = 2.4 Hz, NH), 6.78 (s, 2H, H-1, H-5), 7.48 (t, 4H, *J* = 7.8 Hz), 7.55–7.60 (m, 10H), 7.83 (t, 4H, *J* = 7.8 Hz); ^13^C-NMR (DMSO-*d*_6_): δ = 56.43, 115.79, 119.31, 127.99, 130.01, 131.81, 132.17, 136.96, 138.41, 142.28, 189.38; MS (EI, 70 eV): *m/z* (%) = 643.1 (M^+^+2, 27), 538.1 (6), 471.1 (97), 354.1 (4), 340.1 (36), 171.0 (10), 105.0 (100), 77 (17); HRMS (EI): *m/z* calcd for C_30_H_21_Br_2_N_5_O_2_: 641.0062; found: 641.0048.

*4,8-Di(benzoyl)-2,6-di(2-nitrophenyl)-2,3,6,7,9-pentaazabicyclo[3.3.1]nona-3,7-diene* (**2e**): Pale yellow color; m.p. 233–234 °C; IR (KBr): 3302, 3063, 1664, 1577 cm^−1^; ^1^H-NMR (DMSO-*d*_6_): δ = 4.52 (t, 1H, *J* = 2.4 Hz, NH), 6.71 (d, 2H, *J* = 2.4 Hz, H-1 and H-5), 7.38 (t, 4H, *J* = 7.5 Hz), 7.44 (t, 2H, *J* = 7.8 Hz), 7.50 (t, 2H, *J* = 7.8 Hz), 7.50 (d, 4H, *J* = 7.2 Hz), 7.80–7.86 (m, 4H), 8.00 (d, 2H, *J* = 7.8 Hz); ^13^C-NMR (DMSO-*d*_6_): δ = 58.65, 125.62, 125.74, 126.77, 127.92, 128.93, 131.57, 133.89, 136.93, 137.71, 139.64, 143.16, 189.91; MS (EI, 70 eV): *m/z* (%) = 575.20 (M^+^, 70), 558.2 (48), 527.2 (5), 438.2 (35), 410.2 (10), 305.1 (12), 214.1 (16), 105.0 (100), 77.0 (35); HRMS (EI): *m/z* calcd for C_30_H_21_N_7_O_6_: 575.1553; found: 575.1549.

*4,8-Di(4-chlorobenzoyl)-2,6-diphenyl-2,3,6,7,9-pentaazabicyclo[3.3.1]nona-3,7-diene* (**2f**): Yellow color; m.p. 215–216 °C; IR (KBr): 3337, 3029, 1632, 1589 cm^−1^; ^1^H-NMR (DMSO-*d*_6_): δ = 4.25 (t, 1H, *J* = 2.4 Hz, NH), 6.77 (d, 2H, *J* = 2.4 Hz, H-1 and H-5), 7.11 (t, 2H, *J* = 7.5 Hz), 7.38–7.41 (m, 3H), 7.54–7.57 (m, 3H), 7.64–7.66 (m, 3H), 7.86–7.88 (m, 3H); ^13^C-NMR (DMSO-*d*_6_): δ = 56.46, 117.44, 123.85, 128.04, 129.10, 131.89, 135.80, 136.86, 137.77, 142.98, 188.04; MS (EI, 70 eV): *m/z* (%) = 553.10 (M^+^, 10), 461.0 (36), 414.1 (7), 387.1 (5), 321.0 (4), 296.1 (42), 139.0 (100), 111.0 (23), 77.0 (22); HRMS (EI): *m/z* calcd for C_30_H_21_Cl_2_N_5_O_2_: 553.1072; found: 553.1068.; Crystal Data, C_30_H_21_Cl_2_N_5_O_2_, *M* = 554.42, triclinic, crystal size = 0.140 × 0.260 × 0.370 mm, *a* = 6.3807(7) Å, *b* = 12.9107(13) Å, *c* = 16.2532(17) Å, *α* = 94.517(5)°, *β* = 97.765(5)°, *γ* = 99.214(5)°, *V* = 1302.6(2) Å^3^, *T* = 296(2) K, space group: *P* -1, *Z* = 2, calculated density = 1.414 g/cm^3^, no. of reflection measured 20549, *θ _max_* = 66.87°, *R1* = 0.0557 (CCDC 1885322) [59].

*2,6-Di(4-bromophenyl)-4,8-di(4-chlorobenzoyl)-2,3,6,7,9-pentaazabicyclo[3.3.1]nona-3,7-diene* (**2g**): Orange color; m.p. 261–262 °C; IR (KBr): 3344, 2980, 1635, 1587 cm^−1^; ^1^H-NMR (DMSO-*d*_6_): δ = 4.30 (t, 1H, *J* = 2.4 Hz, NH), 6.76 (d, 2H, *J* = 3 Hz, H-1 and H-5), 7.55 (dd, 4H, *J* =1.8 Hz, *J* = 1.8 Hz), 7.59 (s, 8H), 7.86 (dd, 4H, *J* = 1.8 Hz, J=1.8 Hz); ^13^C-NMR (DMSO-*d*_6_): δ = 56.42, 115.97, 119.43, 128.09, 131.84, 131.96, 135.57, 138.16, 142.22, 188.04; MS (EI, 70 eV): *m/z* (%) = 710.9 (M^+^+2, 4), 540.9 (25), 514.9 (7), 480.9 (3), 437.0 (5), 375.9 (15), 154.9 (15), 139.0 (100), 111.0 (40), 90.0 (5), 75.0 (15); HRMS (EI): *m/z* calcd for C_30_H_19_Br_2_Cl_2_N_5_O_2_: 708.9283; found: 708.9279.

*2,6-Di(4-chlorophenyl)-4,8-di(4-bromobenzoyl)-2,3,6,7,9-pentaazabicyclo[3.3.1]nona-3,7-diene* (**2h**): Pale yallow color; m.p. 255–256 °C; IR (KBr): 3350, 3090, 1634, 1585 cm^−1^; ^1^H-NMR (DMSO-*d*_6_): δ = 4.33 (t, 1H, *J* = 2.4 Hz, NH), 6.76 (d, 2H, *J* = 2.4 Hz, H-1 and H-5), 7.47 (d, 4H, *J* = 9 Hz), 7.66 (d, 4H, *J* = 9 Hz), 7.70 (d, 4H, *J* = 8.4 Hz), 7.80 (d, 4H, *J* = 8.4 Hz); ^13^C-NMR (DMSO-*d*_6_): δ = 56.50, 119.11, 126.13, 127.88, 128.97, 131.05, 132.09, 135.95, 138.09, 141.83, 188.22; MS (EI, 70 eV): *m/z* (%) = 710.7 (M^+^+2, 6), 584.8 (47), 556.8 (3), 387.9 (7), 375.9 (22), 182.9 (100), 154.9 (36), 127.0 (33), 111.0 (26), 75.0 (15); HRMS (EI): *m/z* calcd for C_30_H_19_Br_2_Cl_2_N_5_O_2_: 708.92825; found: 708.9278.

*4,8-Di(4-bromobenzoyl)-2,6-di(4-bromophenyl)-2,3,6,7,9-pentaazabicyclo[3.3.1]nona-3,7-diene* (**2i**): Yellow color; m.p. 264–265 °C; IR (KBr): 3346, 2990, 1638, 1592 cm^−1^; ^1^H-NMR (DMSO-*d*_6_): δ = 4.31 (t, 1H, *J* = 2.7 Hz, NH), 6.73 (d, 2H, *J* = 2.4 Hz, H-1 and H-5), 7.58–7.64 (m, 8H), 7.68 (d, 4H, *J* = 9 Hz), 7.80 (d, 4H, *J* = 8.4 Hz); ^13^C-NMR (DMSO-*d*_6_): δ = 56.43, 115.97, 119.46, 126.15, 131.04, 131.85, 132.10, 135.92, 138.12, 142.22, 188.19; MS (EI, 70 eV): *m/z* (%) = 800.7 (M^+^+4, 2), 630.8 (18), 587.8 (6), 560.8 (12), 480.9 (16), 419.9 (8), 208.9 (12), 182.9 (100), 154.9 (80), 90.0 (12), 76.0 (46); HRMS (EI): *m/z* calcd for C_30_H_19_Br_4_N_5_O_2_: 796.8272; found: 796.8268.

*4,8-Di(4-methoxybenzoyl)-2,6-diphenyl-2,3,6,7,9-pentaazabicyclo[3.3.1]nona-3,7-diene* (**2j**): Pale yellow color; m.p. 237–238 °C; IR (KBr): 3328, 2974, 1626, 1596 cm^−1^; ^1^H-NMR (DMSO-*d*_6_): δ = 3.82 (s, 6H, 2OCH_3_), 4.24 (t, 1H, *J* = 2.4 Hz, NH), 6.76 (d, 2H, *J* = 2.4 Hz, H-1, H-5), 7.03 (dd, 4H, *J* = 1.8 Hz, *J* = 1.8 Hz), 7.08 (t, 2H, *J* = 7.2 Hz), 7.37–7.39 (m, 4H, ArH), 7.64 (d, 4H, *J* = 8.4 Hz), 7.87–7.91 (m, 4H); ^13^C-NMR (DMSO-*d*_6_): δ = 55.41, 56.43, 113.38, 117.10, 123.42, 129.13, 129.45, 132.40, 138.53, 143.13, 162.57, 187.74; MS (EI, 70 eV): *m/z* (%) = 545.0 (M^+^, 11), 453.0 (25), 383.0 (3), 292.0 (22), 135.0 (100), 107.0 (5), 92.0 (12), 77.0 (20); HRMS (EI): *m/z* calcd for C_32_H_27_N_5_O_4_: 545.2063; found: 545.2058.

*2,6-Di(4-chlorophenyl)-4,8-di(4-methoxybenzoyl)-2,3,6,7,9-pentaazabicyclo[3.3.1]nona-3,7-diene* (**2k**): Yellow color; m.p. 247–249 °C; IR (KBr): 3319, 3007, 1597, 1566 cm^−1^; ^1^H-NMR (DMSO-*d*_6_): δ = 3.81 (s, 6H, 2CH_3_O), 4.30 (t, 1H, *J* = 2.4 Hz, NH), 6.76 (d, 2H, *J* = 2.4 Hz, H-1, H-5), 7.03 (d, 4H, *J* = 9 Hz), 7.45 (d, 4H, *J* = 9 Hz), 7.66 (d, 4H, *J* = 9 Hz), 7.91 (d, 4H, *J* = 9 Hz); ^13^C-NMR (DMSO-*d*_6_): δ = 55.40, 56.03, 56.43, 113.18, 113.42, 118.48, 118.71, 118.92, 127.42, 128.96, 132.46, 138.83, 141.97, 162.41, 162.64, 162.84, 187.69; MS (EI, 70 eV): *m/z* (%) = 613.10 (M^+^, 7), 487.1 (25), 450.1 (3), 421.1 (5), 326.1 (10), 161.0 (3), 135.0 (100), 77.0 (10); HRMS (EI): *m/z* calcd for C_32_H_25_Cl_2_N_5_O_4_: 613.1284; found 613.1278. Crystal Data: C_32_H_25_Cl_2_N_5_O_4_, *M* = 614.49, monoclinic, crystal size = 0.200 × 0.120 × 0.020 mm, *a* = 25.381(2) Å, *b* = 7.6990(3) Å, *c* = 25.477(2) Å, *α* = 90°, *β* = 145.50(1)°, *γ* = 90°, *V* = 2819.7(8) Å^3^, *T* = -123.0 °C, space group: *P2*1/c, *Z* = 4, calculated density = 1.447 g/cm^3^, no. of reflection measured 15678, *θ*_max_ = 50.1°, *R1* = 0.0403 (CCDC 1885339) [59].

*2,6-Di(4-bromophenyl)-4,8-di(4-methoxybenzoyl)-2,3,6,7,9-pentaazabicyclo[3.3.1]nona-3,7-diene* (**2l**): Pale yellow color; m.p. 255–256 °C; IR (KBr): 3321, 2968, 1673, 1596 cm^−1^; ^1^H-NMR (DMSO-*d*_6_): δ = 3.82 (s, 6H, 2CH_3_O), 4.28 (t, 1H, *J* = 2.4 Hz, NH), 6.73 (d, 2H, *J* = 2.4 Hz, H-1, H-5), 7.01–7.03 (m, 4H), 7.55–7.59 (m, 8H), 7.88–7.89 (m, 4H); ^13^C-NMR (DMSO-*d*_6_): δ = 55.44, 56.34, 113.45, 115.46, 119.08, 129.22, 131.85, 132.46, 138.84, 142.35, 162.66, 187.68; MS (EI, 70 eV): *m/z* (%) = 702.8 (M^+^+1, 5), 532.9 (20), 371.9 (7), 181.9 (3), 170.9 (6), 135.0 (100), 107.0 (7), 77.0 (13); HRMS (EI): *m/z* calcd for C_32_H_25_Br_2_N_5_O_4_: 701.0273; found: 701.0266.

*2,6-Di(4-chlorophenyl)-4,8-di(4-nitrobenzoyl)-2,3,6,7,9-pentaazabicyclo[3.3.1]nona-3,7-diene* (**2m**): Orange color; m.p. 221–222 °C; IR (KBr): 3329, 2971, 1659, 1599 cm^−1^; ^1^H-NMR (DMSO-*d*_6_): δ = 4.36 (t, 1H, *J* = 2.4 Hz, NH), 6.80 (d, 2H, *J* = 2.4 Hz, H-1 and H-5), 7.46–7.48 (m, 4H), 7.66 (dd, 4H, *J* = 2.4 Hz, *J* = 2.4 Hz), 8.04–8.07 (m, 4H) 8.30–8.32 (m, 4H); ^13^C-NMR (DMSO-*d*_6_): δ = 56.71, 119.43, 122.98, 128.24, 128.95, 131.18, 137.88, 141.73, 142.64, 149.04, 162.24, 187.98; MS (EI, 70 eV): *m/z* (%) = 642.7 (M^+^, 4), 516.8 (18), 464.8 (16), 435.9 (22), 401.9 (20), 352.9 (16), 302.9 (12), 176.0 (10), 150.0 (100), 110.9 (58), 76.0 (32); HRMS (EI): *m/z* calcd for C_30_H_19_Cl_2_N_7_O_6_: 643.0774; found: 645.0741.

*2,6-Di(4-bromophenyl)-4,8-di(4-nitrobenzoyl)-2,3,6,7,9-pentaazabicyclo[3.3.1]nona-3,7-diene* (**2n**): Yellow color; m.p. 271–272 °C; IR (KBr): 3329, 3073, 1650, 1601 cm^−1^; ^1^H-NMR (DMSO-*d*_6_): δ = 4.36 (t, 1H, *J* = 2.4 Hz, NH), 6.80 (d, 2H, *J* = 3 Hz, H-1 and H-5), 7.59–7.63 (m, 8H), 8.06 (d, 4H, *J* = 8.4 Hz), 8.31 (d, 4H, *J* = 9 Hz); ^13^C-NMR (DMSO-*d*_6_): δ = 56.65, 116.38, 119.80, 123.03, 131.23, 131.89, 137.93, 142.15, 142.65, 149.07, 162.28, 188.02; MS (EI, 70 eV): *m/z* (%) = 732.7 (M^+^, 6), 562.8 (36), 532.8 (21), 509.8 (8), 479.8 (14), 398.9 (20), 386.9 (13), 243.0 (10), 181.9 (23), 170.9 (37), 150.0 (100), 120.0 (22), 104.0 (63), 76.0 (56); HRMS (EI): *m/z* calcd for C_30_H_19_Br_2_N_7_O_6_: 732.9744; found: 732.9739.

*4,8-Diacetyl-2,6-di(4-chlorophenyl)-2,3,6,7,9-pentaazabicyclo[3.3.1]nona-3,7-diene* (**2o**): Pale yellow color; m.p. 228–230 °C; IR (KBr): 3248, 2995, 1656, 1593 cm^−1^; ^1^H-NMR (DMSO-*d*_6_): δ = 2.33 (s, 6H, 2CH_3_), 4.05 (t, 1H, *J* = 2.4 Hz, NH), 6.41 (d, 2H, *J* = 2.4 Hz, H-1 and H-5), 7.47 (d, 4H, *J* = 9 Hz), 7.79 (d, 4H, *J* = 9 Hz); ^13^C-NMR (DMSO-*d*_6_): δ = 24.22, 55.99, 119.08, 127.54, 128.69, 139.15, 141.85, 194.72; MS (EI, 70 eV): *m/z* (%) = 429.2 (M^+^,28), 386.1 (14), 359.1 (7), 332.1 (3), 303.1(100), 261.1 (30), 234.1 (77), 198.1 (15), 138.0 (36), 111.0 (57), 75.0 (12); HRMS (EI): *m/z* calcd for C_20_H_17_Cl_2_N_5_O_2_: 429.0760; found: 429.0752.

*4,8-Diacetyl-2,6-di(4-bromophenyl)-2,3,6,7,9-pentaazabicyclo[3.3.1]nona-3,7-diene* (**2p**): Yellow color; m.p. 236–237 °C; IR (KBr): 3245, 3006, 1655, 1581 cm^−1^; ^1^H-NMR (DMSO-*d*_6_): δ = 2.33 (s, 6H, 2CH_3_), 4.06 (t, 1H, *J* = 2.4 Hz, NH), 6.40 (s, 2H, H-1 and H-5), 7.59–7.58 (m, 4H), 7.74–7.72 (m, 4H); ^13^C-NMR (DMSO-*d*_6_): δ = 24.29, 55.92, 115.64, 119.46, 131.63, 139.19, 142.25, 194.78; MS (EI, 70 eV): *m/z* (%) = 519.1 (M^+^, 44), 476.1 (20), 449.1 (10), 422.0 (5), 347.1 (100), 305.1 (27), 280.1 (70), 266.1 (10), 182.0 (25), 157.0 (33), 143.0 (3), 91.1 (8); HRMS (EI): *m/z* calcd for C_20_H_17_Br_2_N_5_O_2_: 518.9729; found: 518.9727. Crystal Data, C_20_H_17_Br_2_N_5_O_2_, *M* = 519.19, monoclinic, crystal size = 0.130 × 0.090 × 0.020 mm, *a* = 11.766(2) Å, *b* = 10.114(2) Å, *c* = 17.408(3) Å, *α* = 90°, *β* = 103.573(8) °, *γ* = 90°, *V* = 2013.8(6)Å^3^, *T* = 20.0 °C, space group: *P*2_1_, Z = 4, calculated density = 1.712 g/cm^3^, no. of reflection measured 11322, *θ_max_* = 51.1°, *R1* = 0.0862 (CCDC 1888859) [59].

## 4. Conclusions

A new series of C*_2_*-symmetric 2,3,6,7,9-pentaazabicyclo[3.3.1]nonane derivatives were synthesized in high yields through one-pot double Mannich-type reaction of Et_3_N-catalyzed 3-oxo-2-arylhydrazonopropanals with double equivalents of ammonium acetate under three different heating platforms: conventional, ultrasound and microwave irradiation. Single crystal X-ray analysis supported the elucidation of the structures of the obtained products.

## Figures and Tables

**Table 1:**
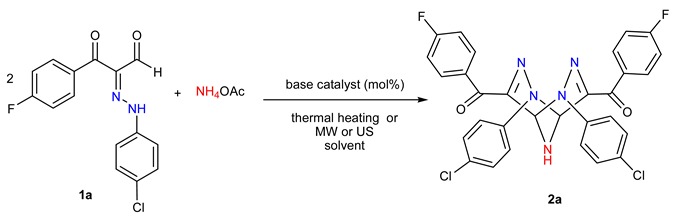
Optimization the dimerization condition of 3-oxo-2-arylhydrazonopropanals **1a** with ammonium acetate *^a.^*

Run	Base-Catalyst (mol%)	Solvent	Conv. Heating		Sonication		MW Irradiation	
			Yield *^b^* %	Time (h)	Yield *^b^* %	Time (min)	Yield *^b^* %	Time (min)
1	No catalyst	EtOH	trace	15	trace	120	trace	30
2	Et_3_N (15)	EtOH	70	4	82	60	87	5
3	Et_3_N (25)	EtOH	78 *^c^*	3	89 *^d^*	50	94	3
4	Et_3_N (30)	EtOH	71	3	85	60	90	4
5	Et_3_N (25) *^e^*	EtOH	66 *^e^*	3	73 *^e^*	50	80*^e^*	3
6	Et_3_N (25) *^f^*	EtOH	60 *^f^*	3	68 *^f^*	50	73*^f^*	3
7	Et_3_N (25)	MeOH	71	4	82	50	89	4
8	Et_3_N (25)	isopropanol	70	4	80	60	88	4
9	Et_3_N (25)	*n*-hexane	34	6	52	80	63	10
10	Et_3_N (25)	acetic acid	32	4	40	70	50	6
11	Et_3_N (25)	DMF	30	6	45	100	55	10
12	Et_3_N (25)	toluene	35	5	50	90	65	10
13	pyridine (25)	EtOH	20	4	28	60	35	5
14	DABCO (25)	EtOH	17	5	20	80	28	7
15	DBU (25)	EtOH	15	5	18	70	25	8
16	NaHCO_3_ (25)	EtOH	10	6	10	80	15	10
17	K_2_CO_3_ (25)	EtOH	10	5	12	90	15	10
18	NaOH (25)	EtOH	12	5	14	80	18	9

*^a^ Reaction conditions:* Arylhydrazonopropanal **1a** (5 mmol), ammonium acetate (10 mmol) and base-catalyst (15~30 mol%) in solvent (7 mL) at reflux temperature for conventional heating 3~6 h, ultrasonic irradiation at 80 °C (110 W) for 50~100 min, or microwave irradiation at 80 °C (200 W) for 3~10 min. *^b^* isolated yield. *^c^* Yield was 20% after 50 min. *^d^* Yield was 35% after 15 min. *^e^* Compound **1a** (5 mmol) and ammonium acetate (5 mmol) were used. *^f^* Compound **1a** (10 mmol) and ammonium acetate (5 mmol) were used. Conv. = conventional, MW = microwave.

**Table 2:**
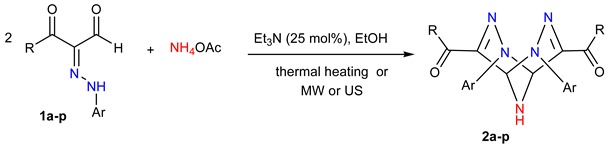
Et_3_N-catalyzed synthesis of 2,3,6,7,9-pentaazabicyclo[3.3.1]nona-3,7-dienes **2a**–**p**.

Run	Products	R	Ar	Conv. Heating *^a^*		Sonication *^a^*		MW *^a^* Irradiation	
				Yield *^b^*%	Time (h)	Yield *^b^* %	Time (min)	Yield *^b^* %	Time (min)
1	**2a**	4-FC_6_H_4_	4-ClC_6_H_4_	78	3	89	50	94	3
2	**2b**	4-FC_6_H_4_	4-BrC_6_H_4_	77	4	87	60	93	4
3	**2c**	C_6_H_5_	4-ClC_6_H_4_	70	3	81	50	89	4
4	**2d**	C_6_H_5_	4-BrC_6_H_4_	72	5	82	70	90	6
5	**2e**	C_6_H_5_	2-NO_2_C_6_H_4_	68	4	80	70	86	5
6	**2f**	4-ClC_6_H_4_	C_6_H_5_	71	3	83	40	90	3
7	**2g**	4-ClC_6_H_4_	4-BrC_6_H_4_	73	5	84	70	91	5
8	**2h**	4-BrC_6_H_4_	4-ClC_6_H_4_	86	4	87	70	92	5
9	**2i**	4-BrC_6_H_4_	4-BrC_6_H_4_	84	5	86	80	92	6
10	**2j**	4-OMeC_6_H_4_	C_6_H_5_	66	5	77	70	82	5
11	**2k**	4-OMeC_6_H_4_	4-ClC_6_H_4_	65	6	77	90	81	6
12	**2l**	4-OMeC_6_H_4_	4-BrC_6_H_4_	67	8	78	100	83	7
13	**2m**	4-NO_2_C_6_H_4_	4-ClC_6_H_4_	67	7	79	110	84	7
14	**2n**	4-NO_2_C_6_H_4_	4-BrC_6_H_4_	71	8	80	100	86	8
15	**2o**	CH_3_	4-ClC_6_H_4_	62	7	73	90	81	8
16	**2p**	CH_3_	4-BrC_6_H_4_	64	6	75	110	81	9

*^a^ Reaction conditions:* 3-Oxo-2-arylhydrazonopropanals **1a**–**p** (5 mmol), ammonium acetate (10 mmol) and Et_3_N (25 mol%) in EtOH (7 mL) at reflux temperature for conventional heating 3~8 h, ultrasonic irradiation at 80 °C (110 W) for 50~110 min, or microwave irradiation at 80 °C (200 W) for 3~9 min. *^b^* Isolated yields. Conv. = conventional, MW = microwave.

## Supplementary Materials

Supplementary materials (^1^H and ^13^C-NMR spectral sheets) are available online.
