# Supplementary material for: Facile Assembling of Novel 2,3,6,7,9-pentaazabicyclo- [3.3.1]nona-3,7-diene Derivatives under Microwave and Ultrasound Platforms
Source: Molecules. 2019 Mar 20;24(6):1110. doi: 10.3390/molecules24061110 (PMC6471913; doi:10.3390/molecules24061110)

Supporting Information on

Facile assembling of novel 2,3,6,7,9-pentaazabicyclo[3.3.1]nona-3,7-diene derivatives under microwave and ultrasound platforms

Hamad M. Al-Matar[a*], Kamal M. Dawood[b], Wael M. Tohamy[c] and Mona A. Shalaby[a]

^{1}H NMR spectrum of compound **2a**

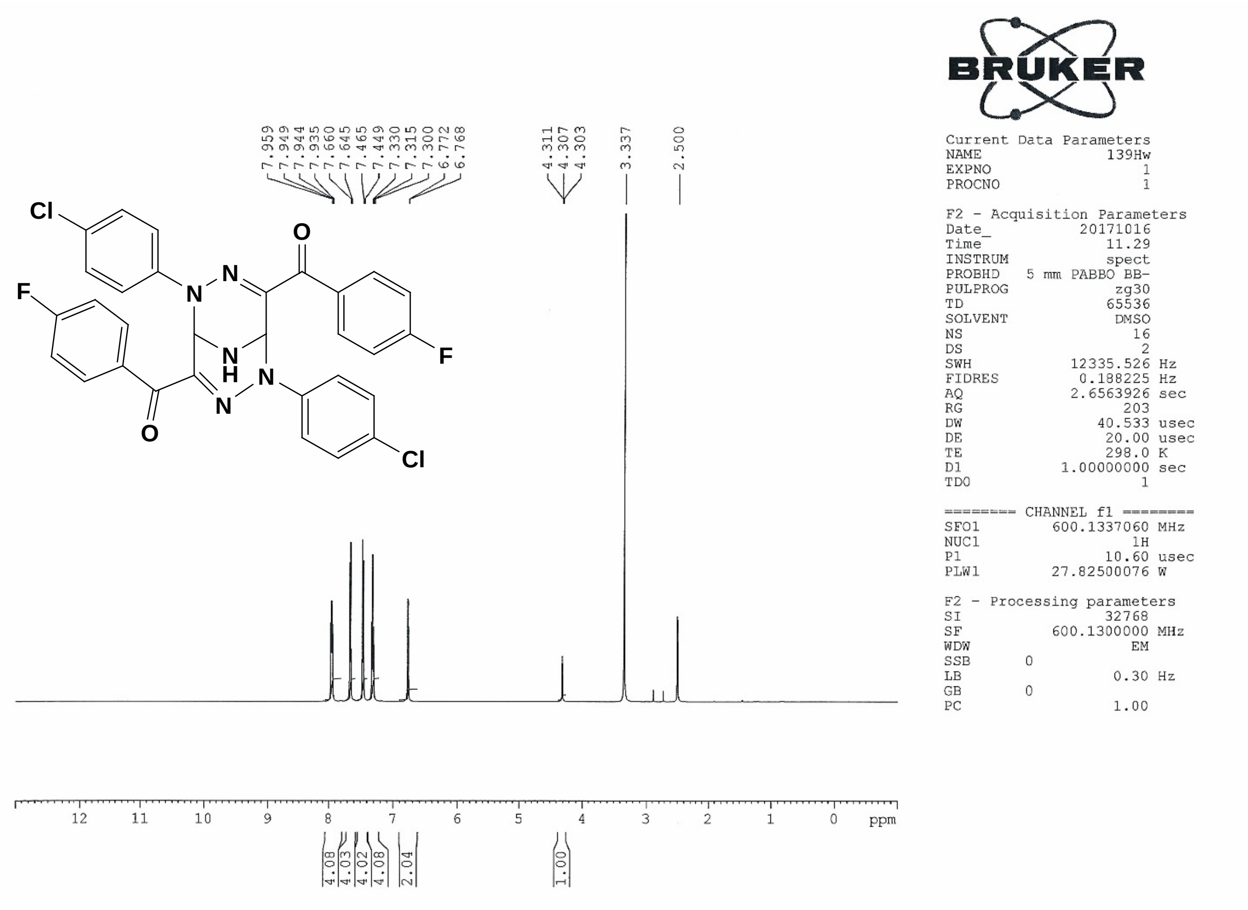

^{13}C NMR spectrum of compound **2a**

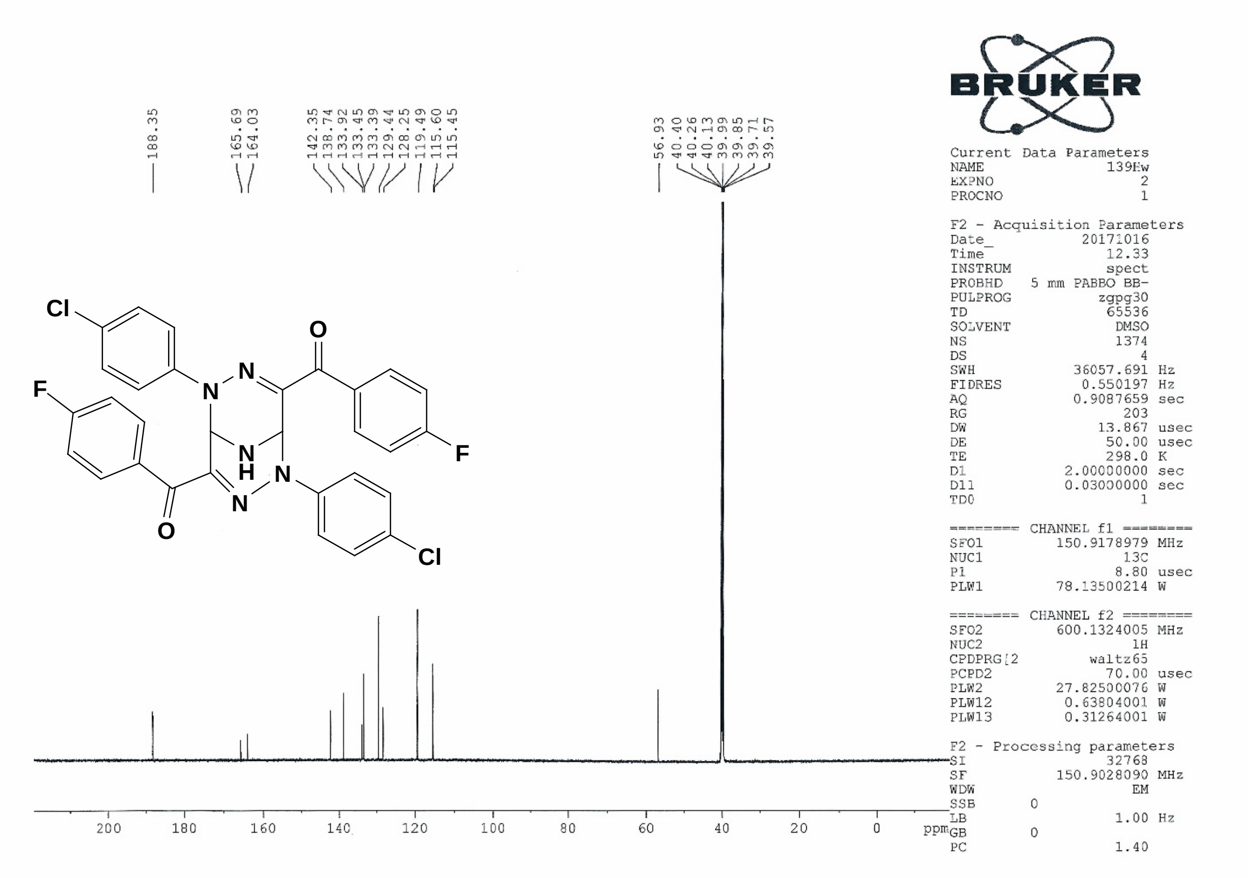

^{1}H NMR spectrum of compound **2b**

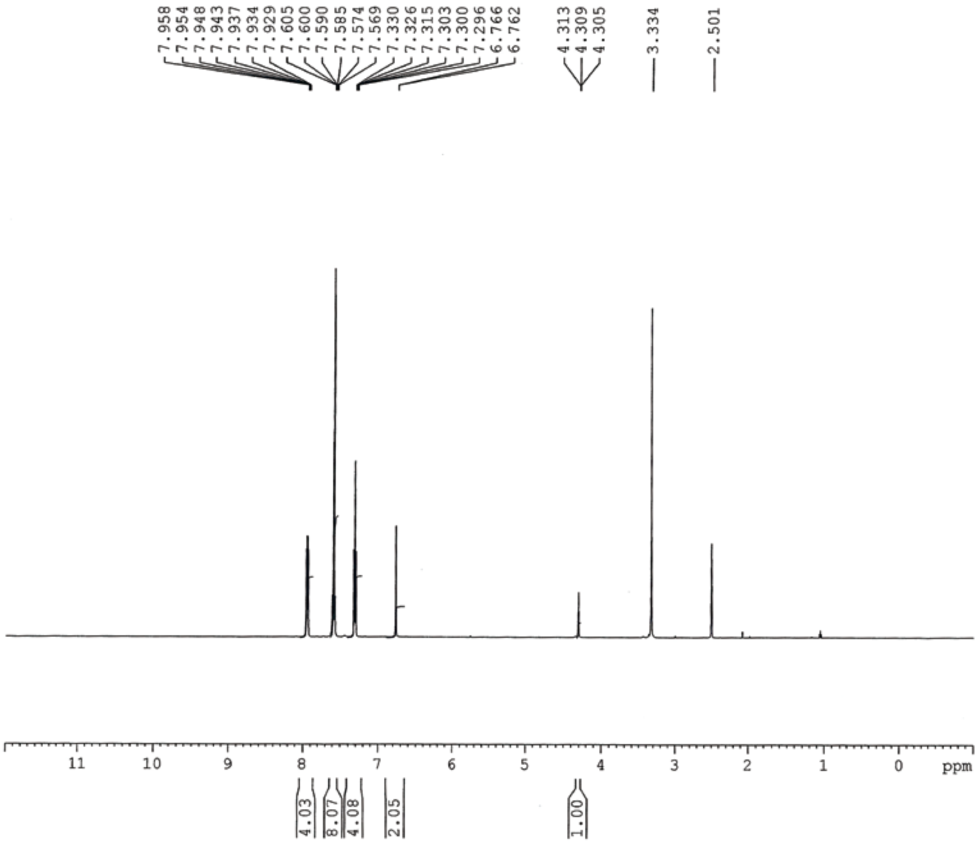


```
Current Data Parameters
NAME              137HW-1H
EXPNO                    1
PROCNO                   1

F2 - Acquisition Parameters
Date_             20171015
Time                 12.36
INSTRUM              spect
PROBHD    5 mm PABBO BB-
PULPROG               zg30
TD                   65536
SOLVENT               DMSO
NS                      16
DS                       2
SWH             12335.526 Hz
FIDRES           0.188225 Hz
AQ              2.6563926 sec
RG                     203
DW                  40.533 usec
DE                   20.00 usec
TE                   298.0 K
D1             1.00000000 sec
TD0                      1

======== CHANNEL f1 ========
SFO1        600.1337060 MHz
NUC1                    1H
P1                   10.60 usec
PLW1          27.82500076 W

F2 - Processing parameters
SI                   32768
SF          600.1300000 MHz
WDW                     EM
SSB       0
LB                    0.30 Hz
GB        0
PC                    1.00
```

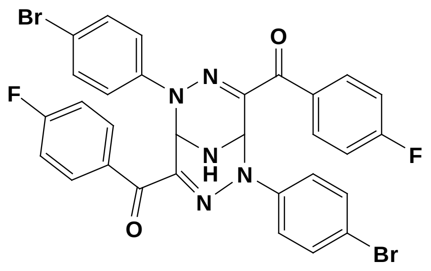

^{13}C NMR spectrum of compound **2b**

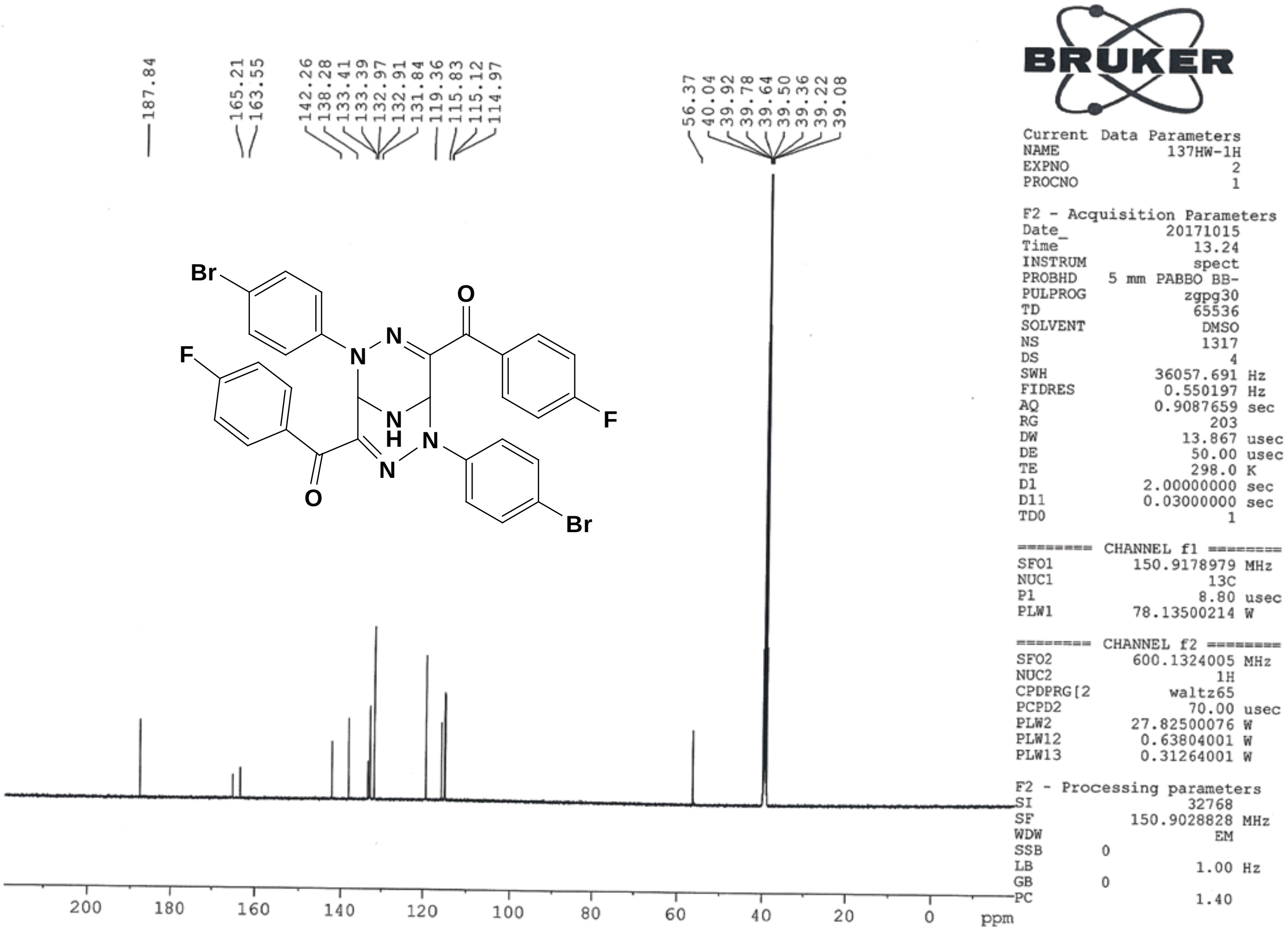

^{1}H NMR spectrum of compound **2c**

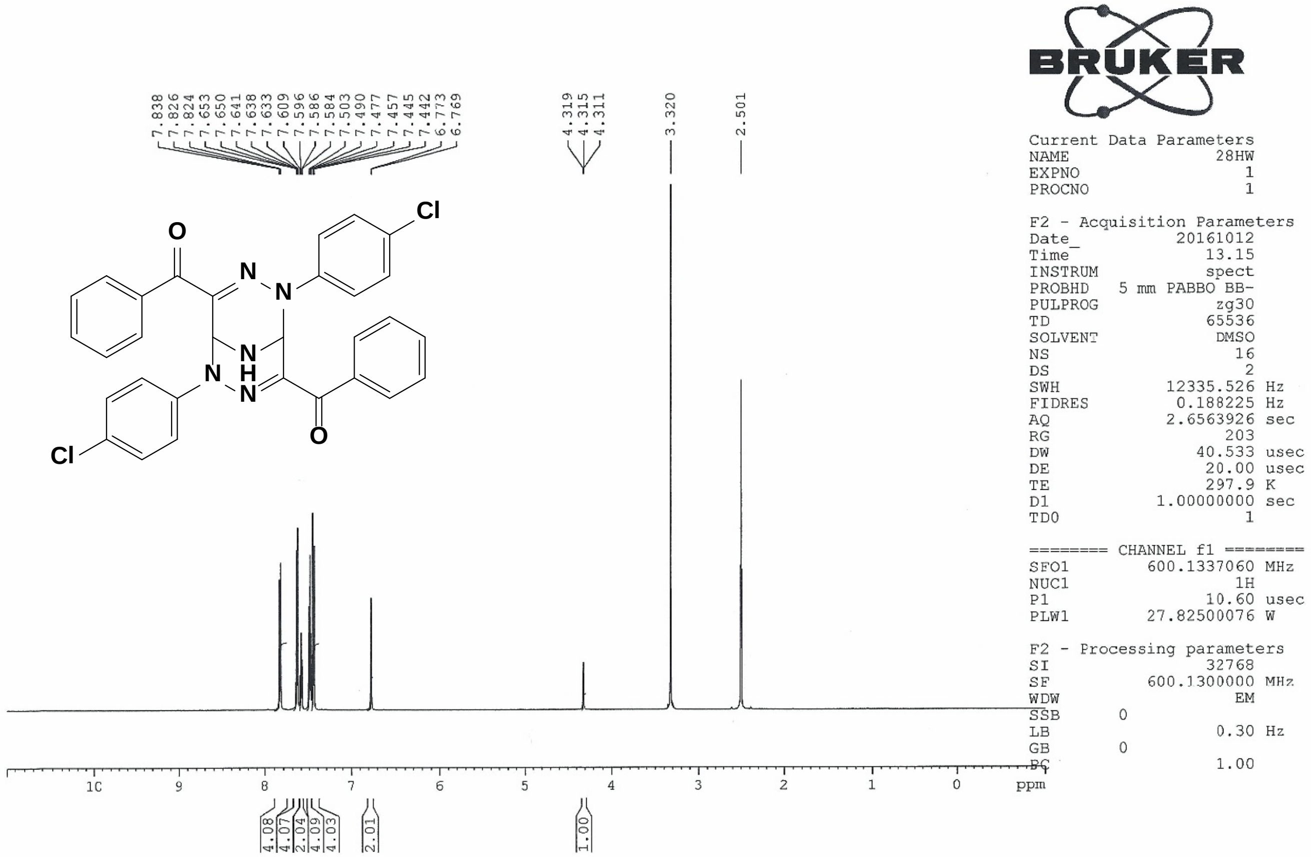

^{13}C NMR spectrum of compound **2c**

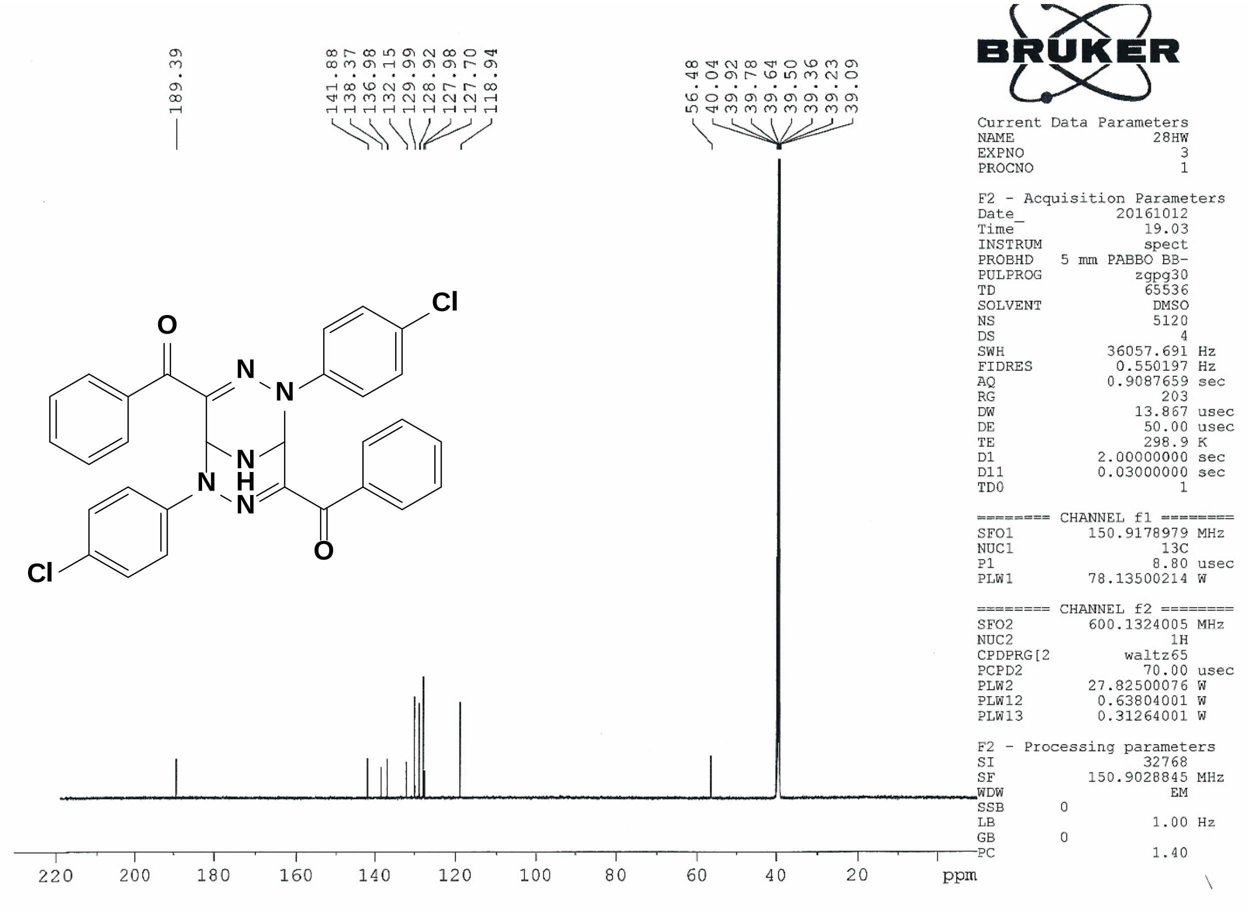

^{1}H NMR spectrum of compound **2d**

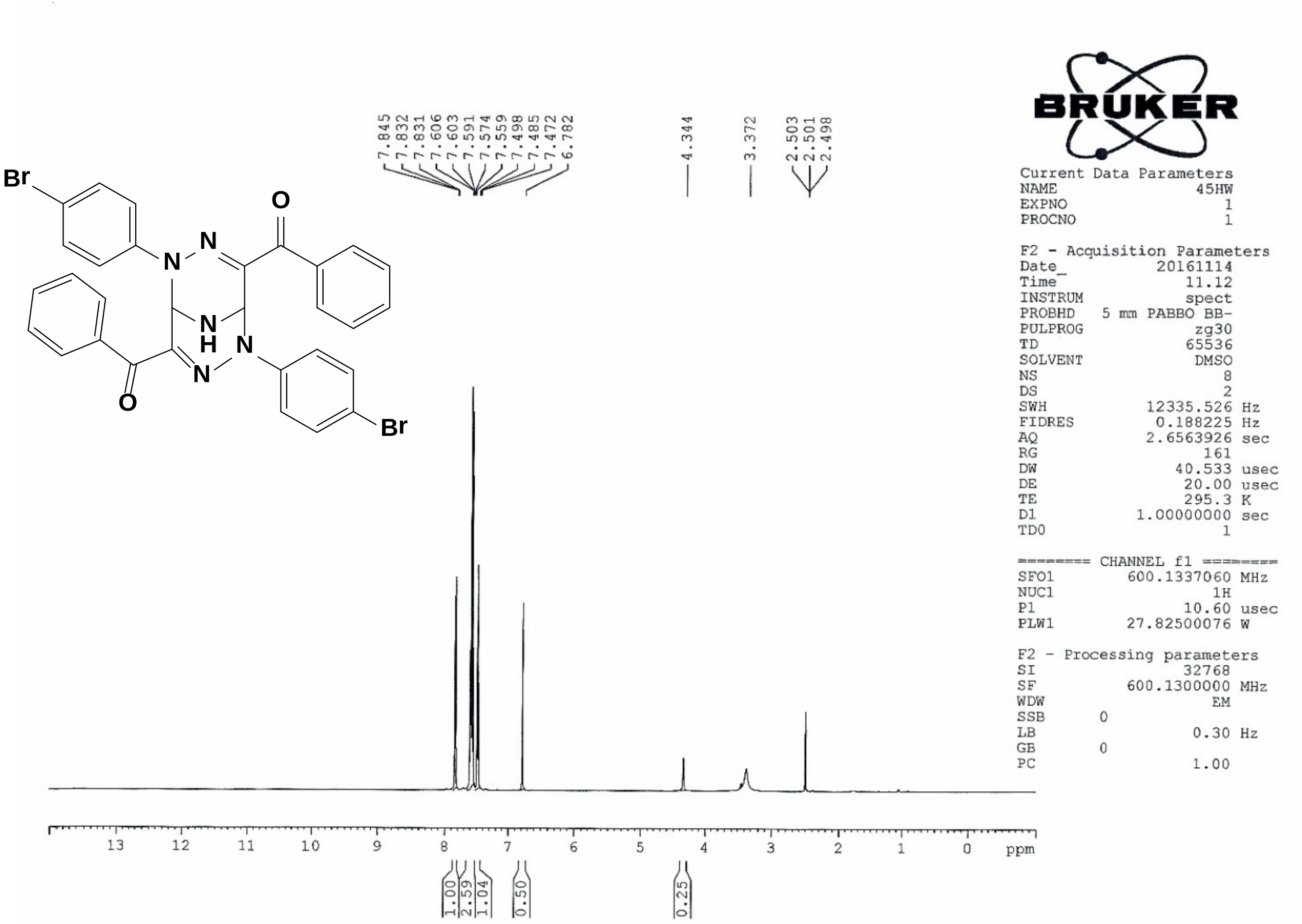

^{13}C NMR spectrum of compound **2d**

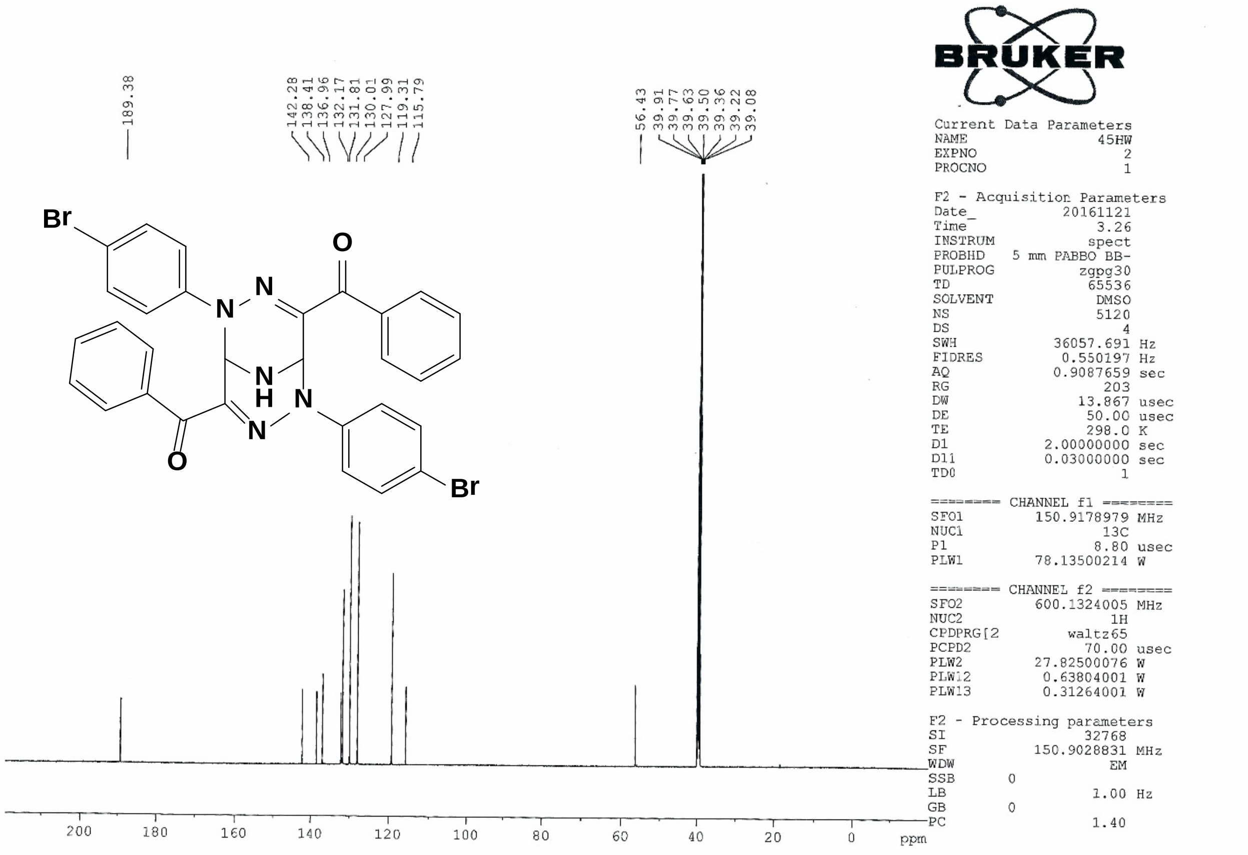

^{1}H NMR spectrum of compound **2e**

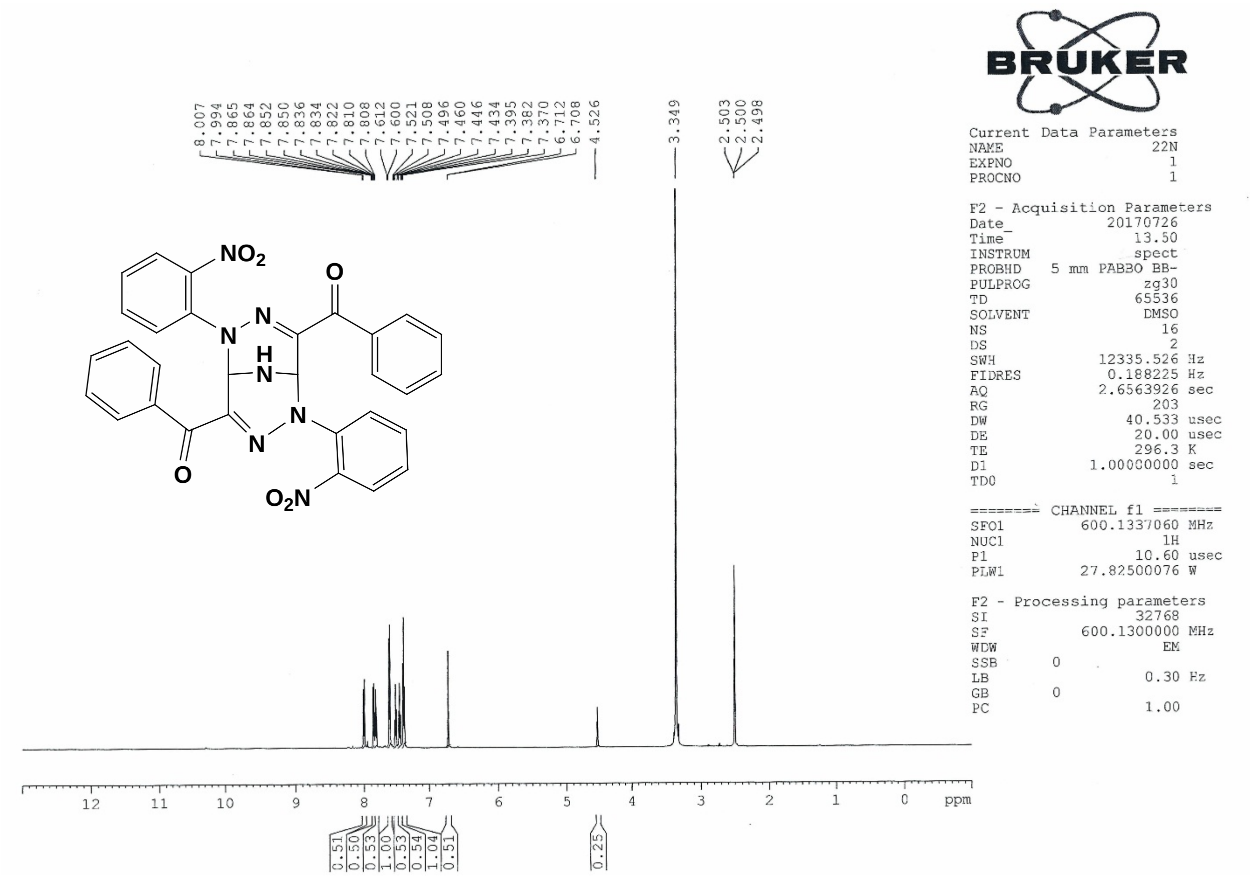

^{13}C NMR spectrum of compound **2e**

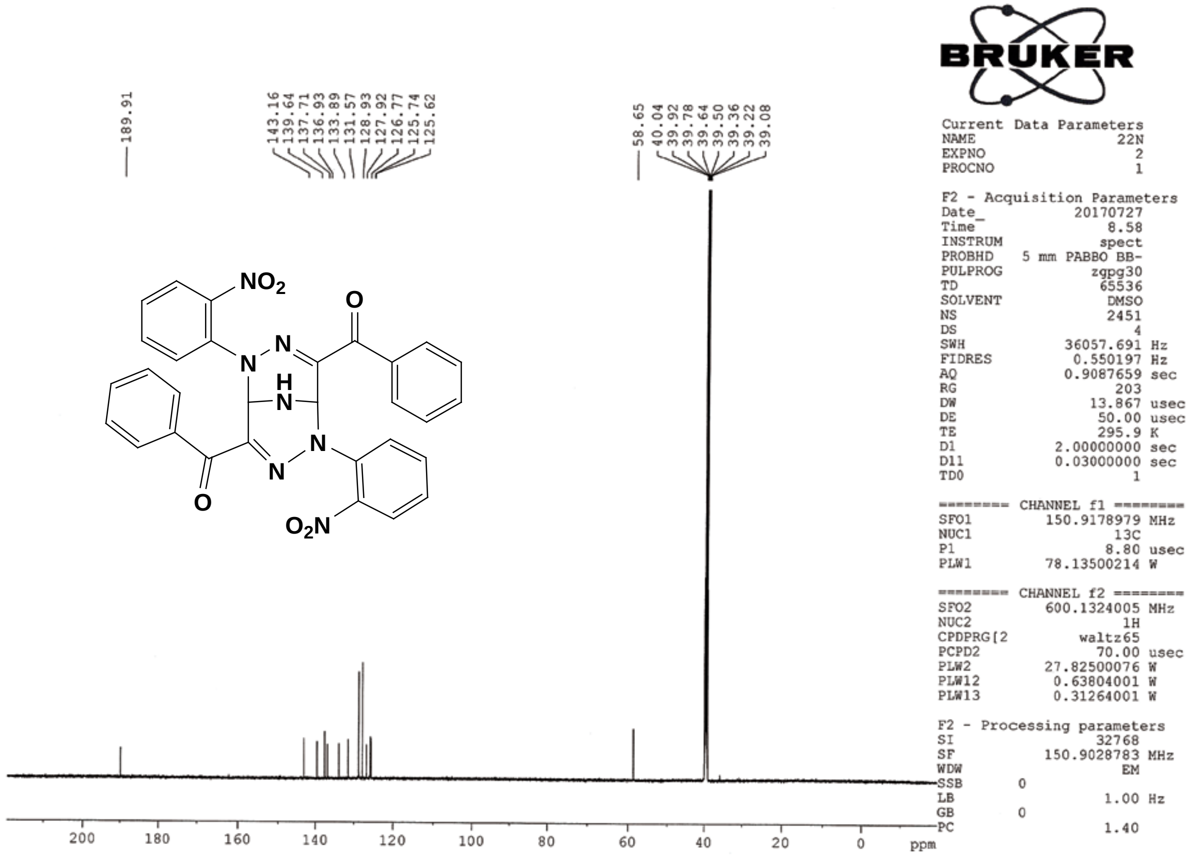

^{1}H NMR spectrum of compound **2f**

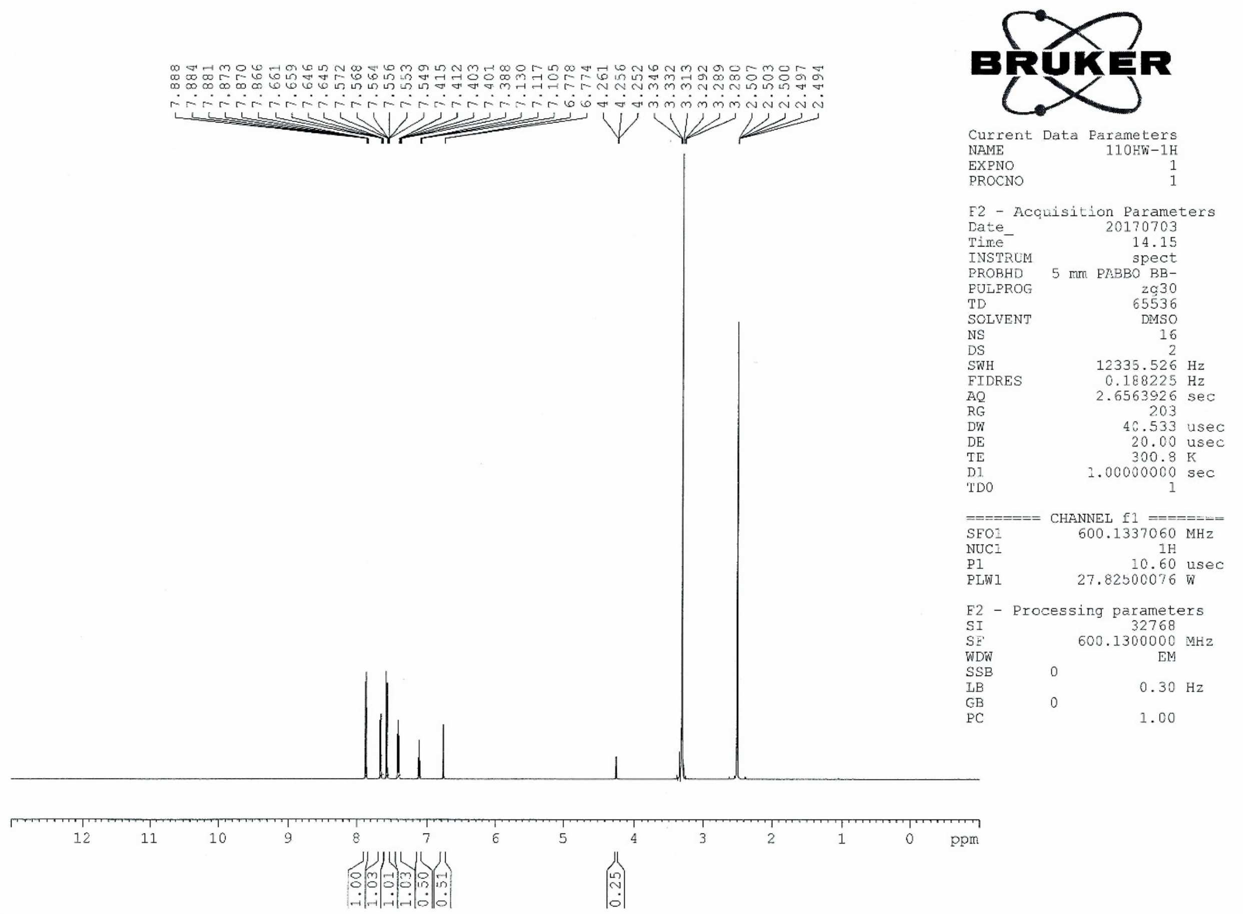

^{13}C NMR spectrum of compound **2f**

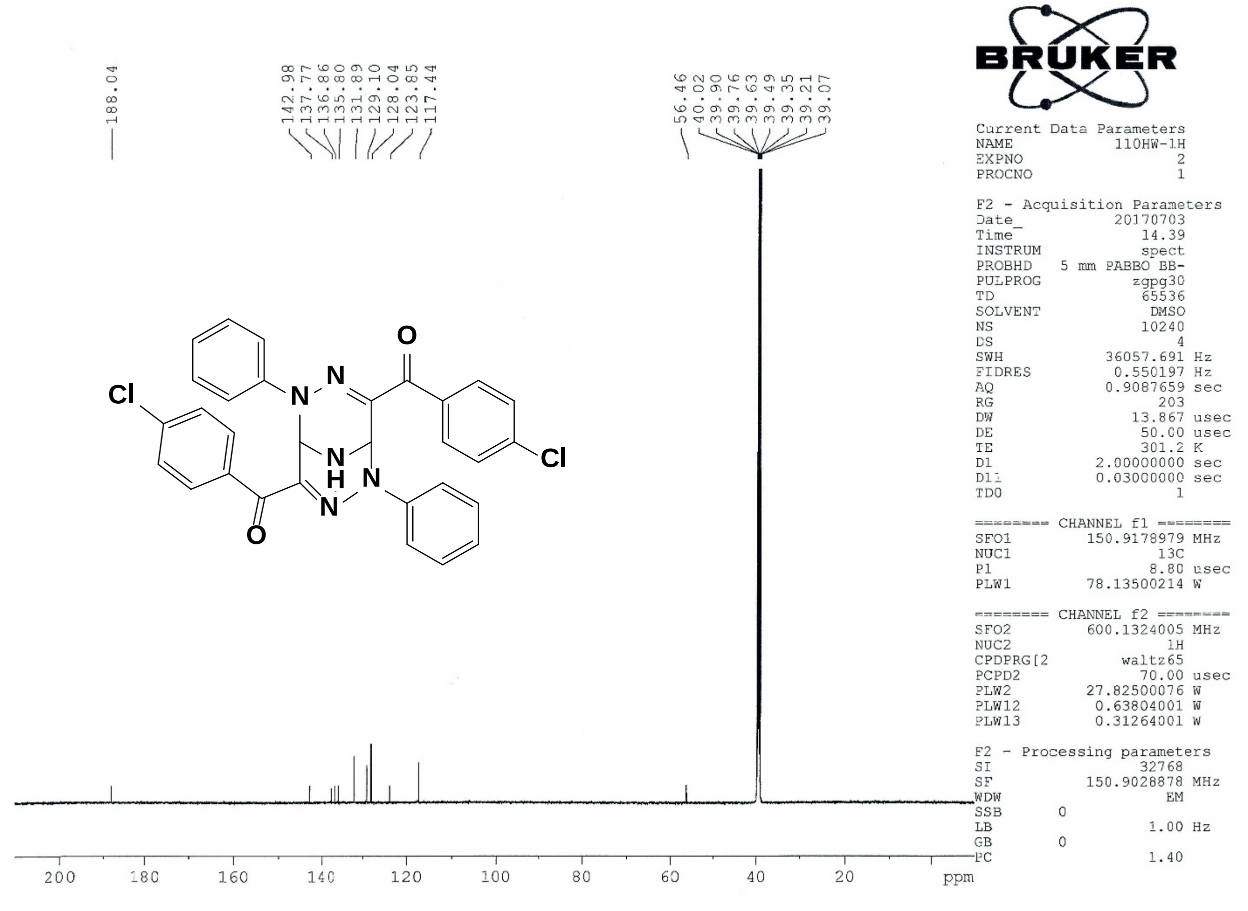

^{1}H NMR spectrum of compound **2g**

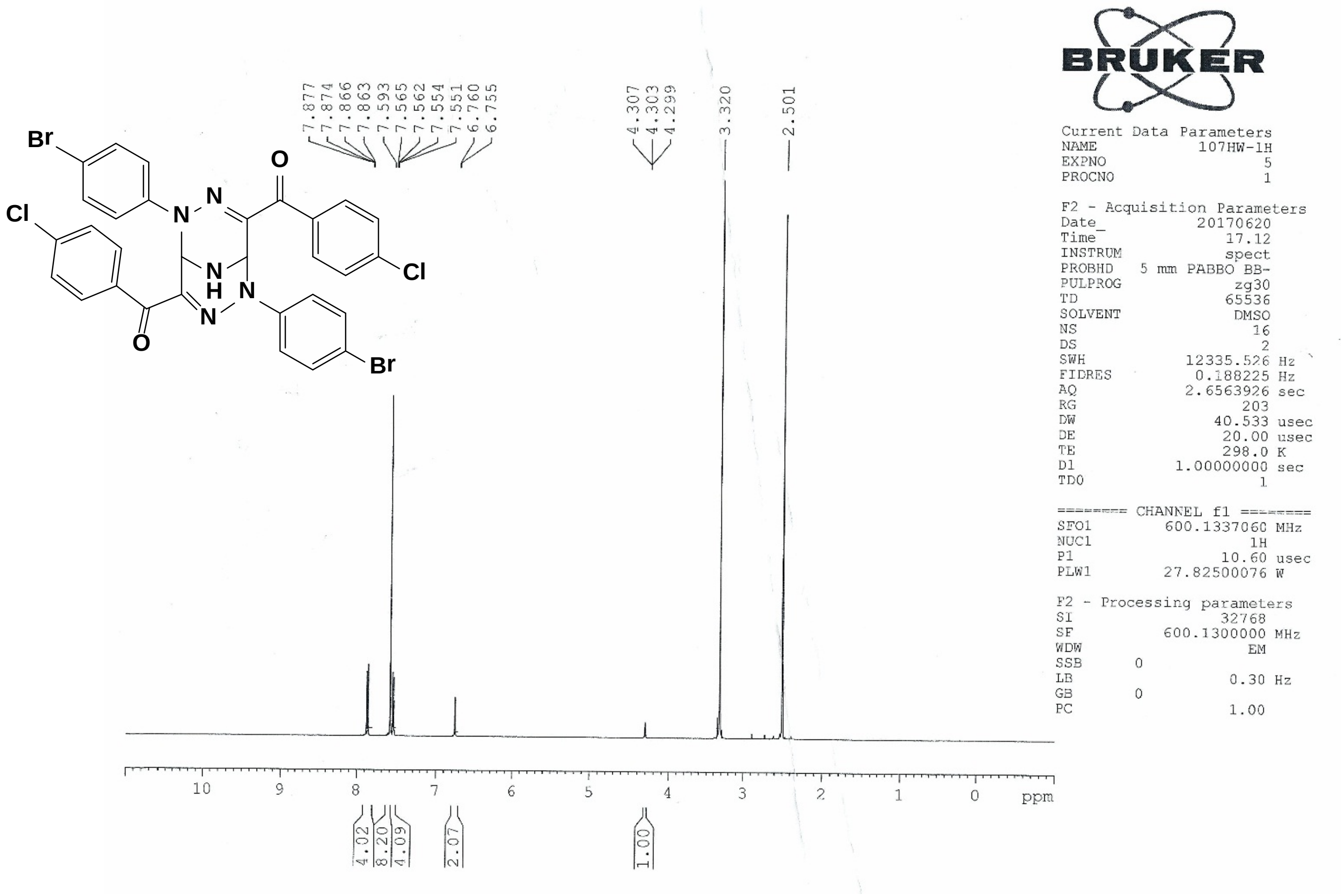

^{13}C NMR spectrum of compound **2g**

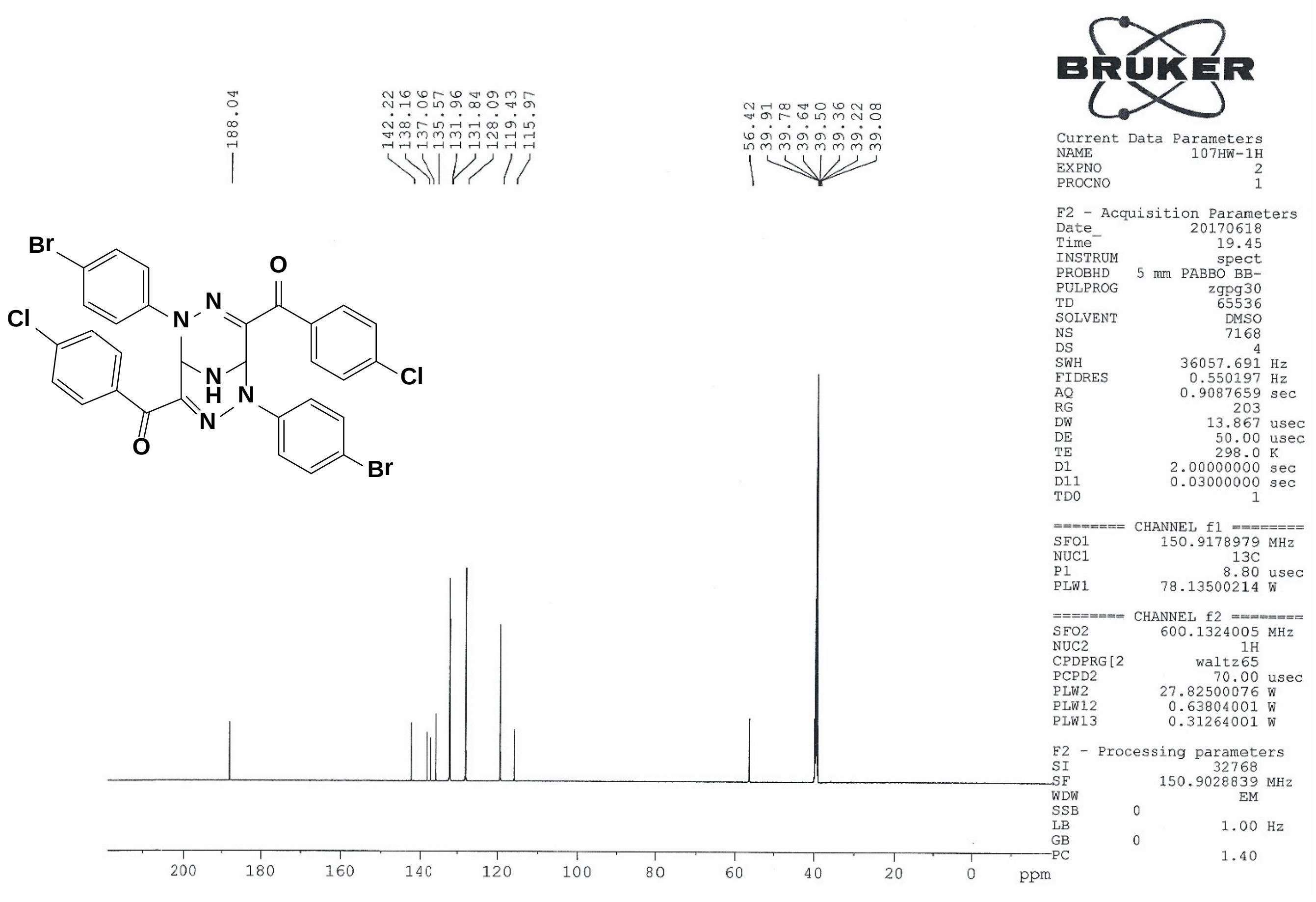

^{1}H NMR spectrum of compound **2h**

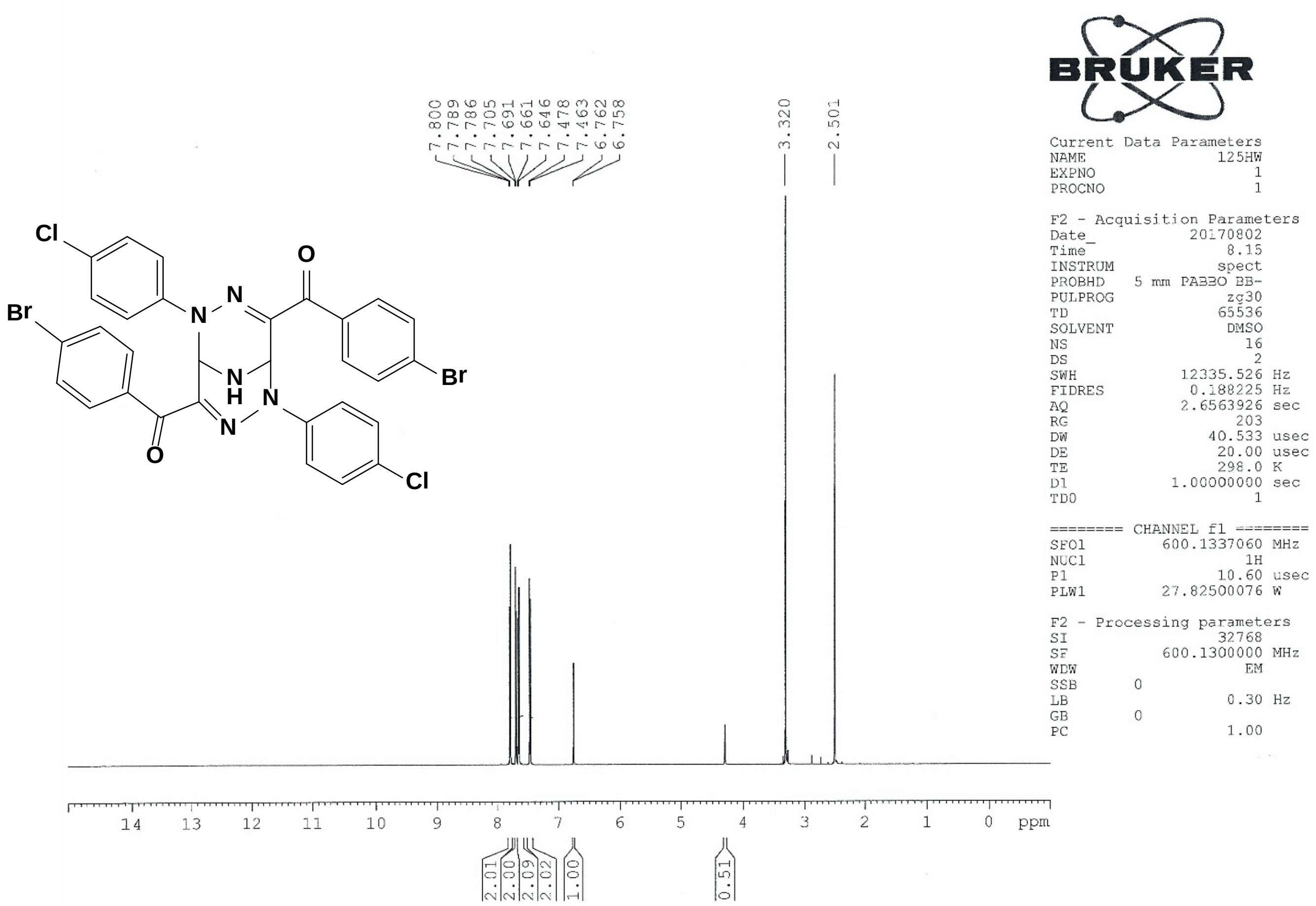

^{13}C NMR spectrum of compound **2h**

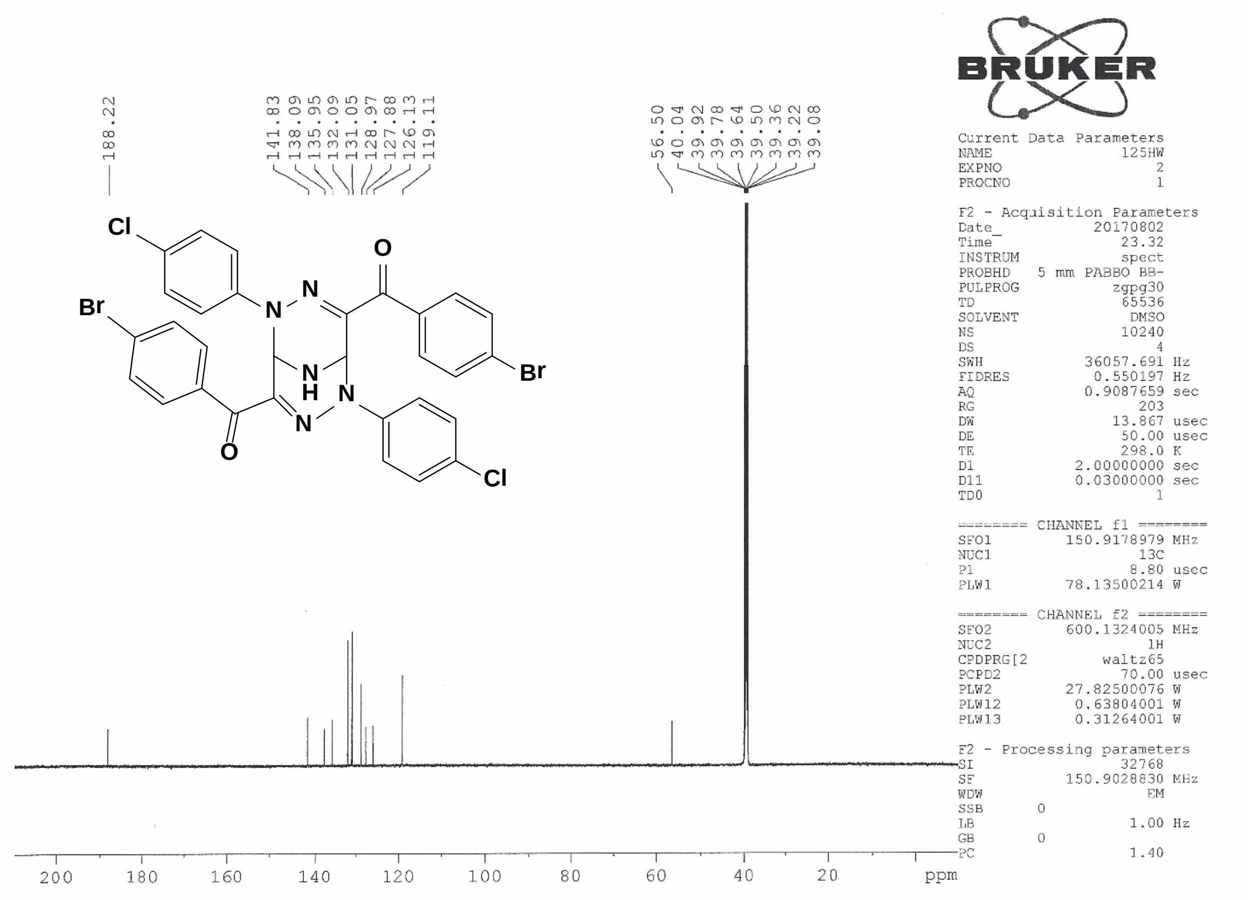

^{1}H NMR spectrum of compound **2i**

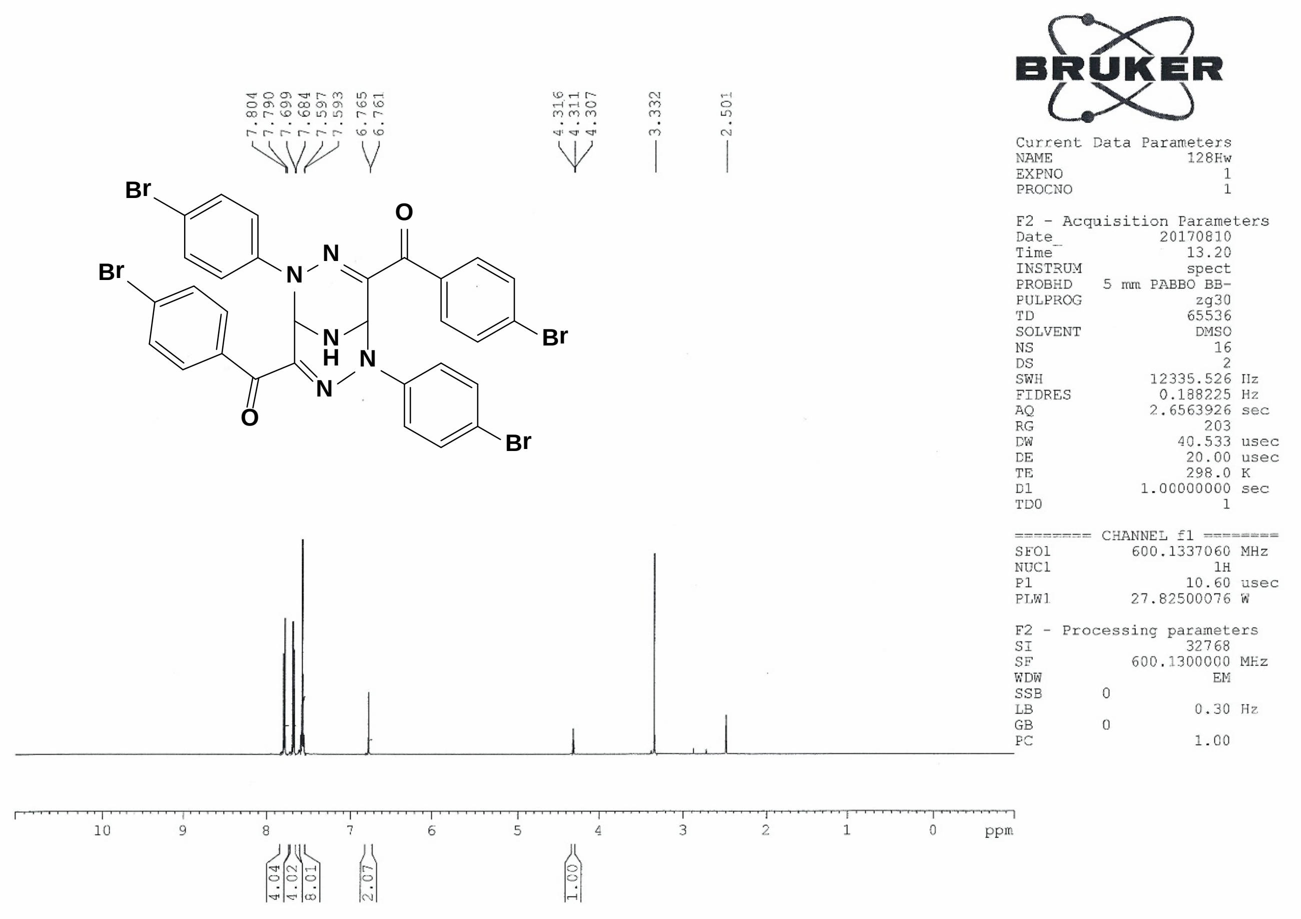

^{13}C NMR spectrum of compound **2i**

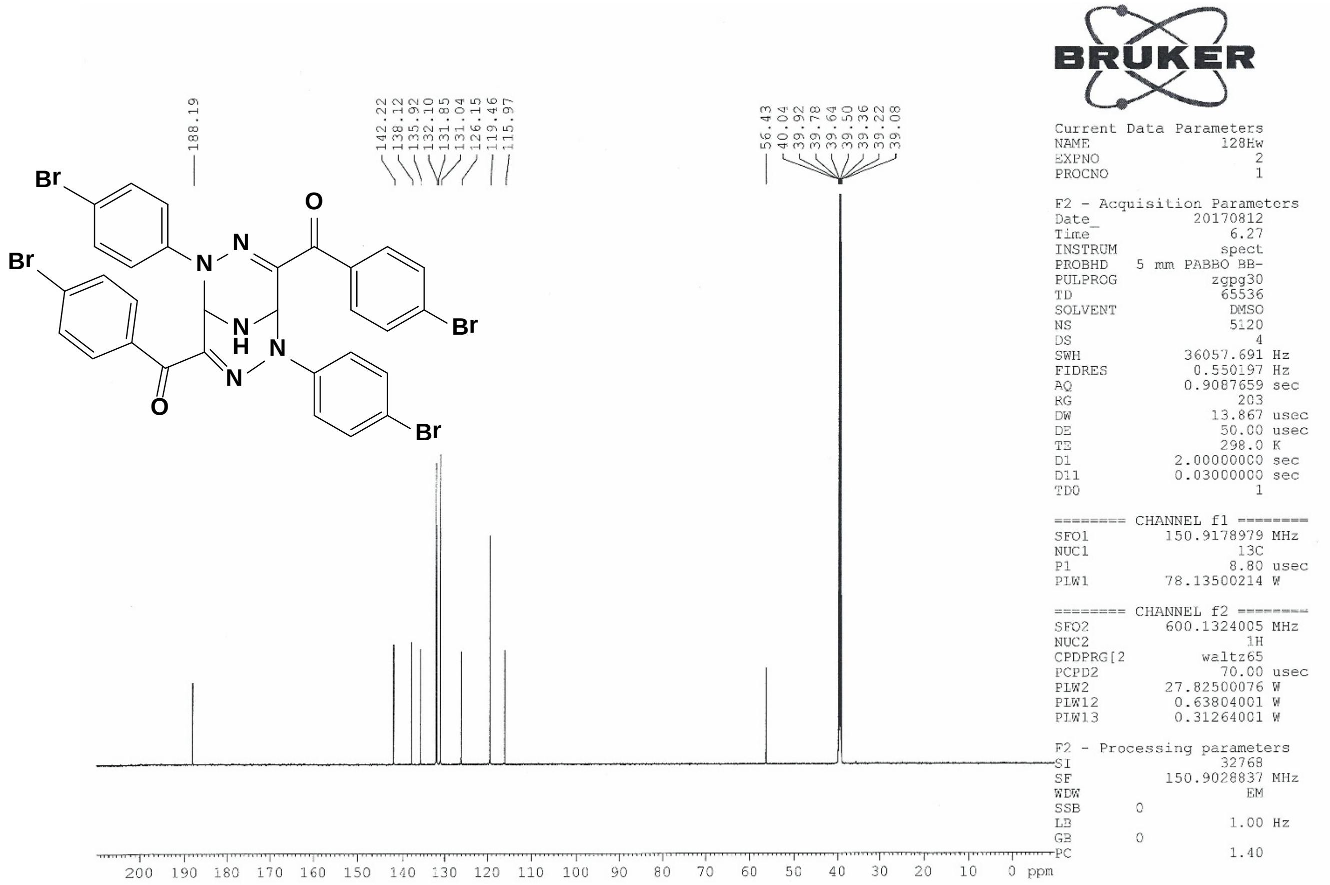

^{1}H NMR spectrum of compound **2j**

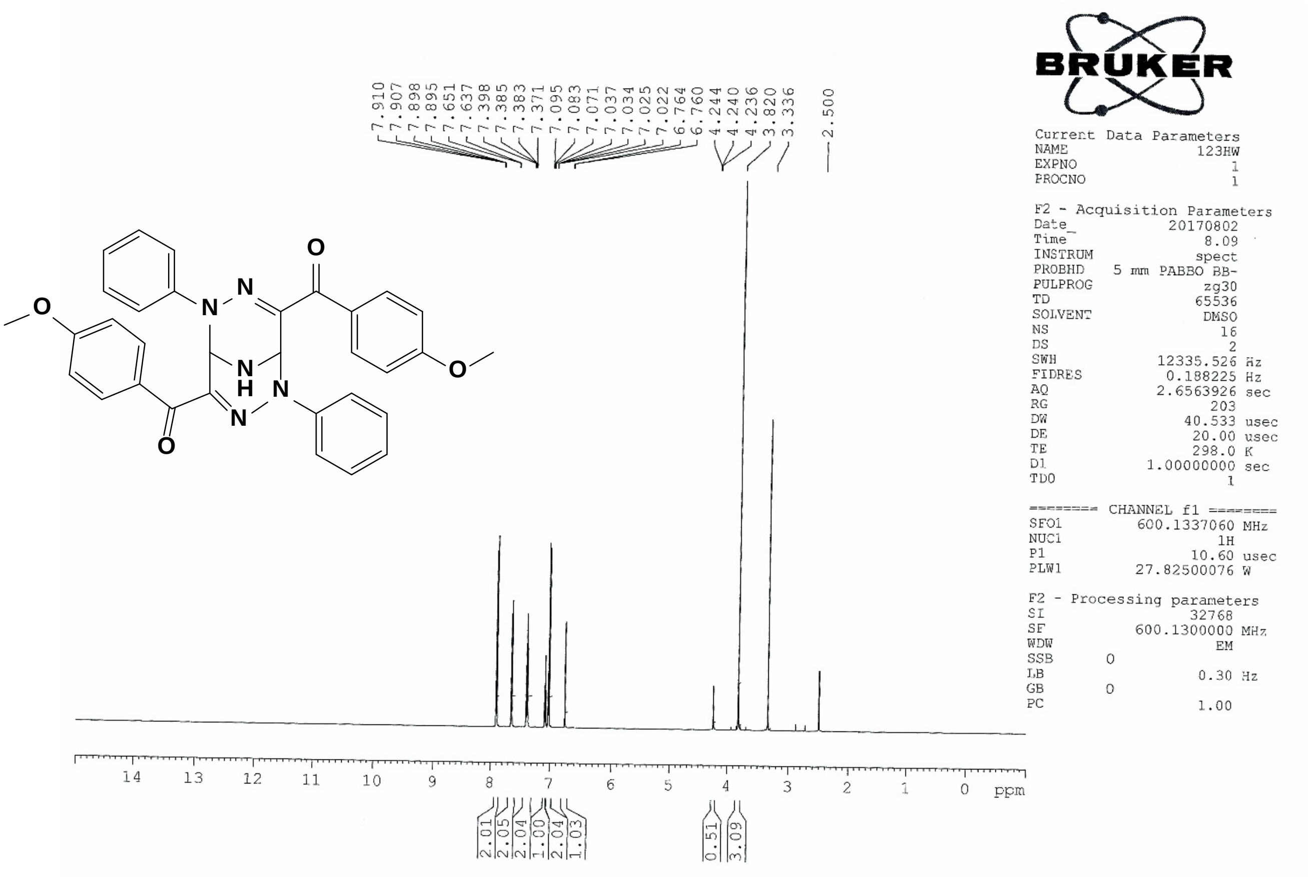

^{13}C NMR spectrum of compound **2j**

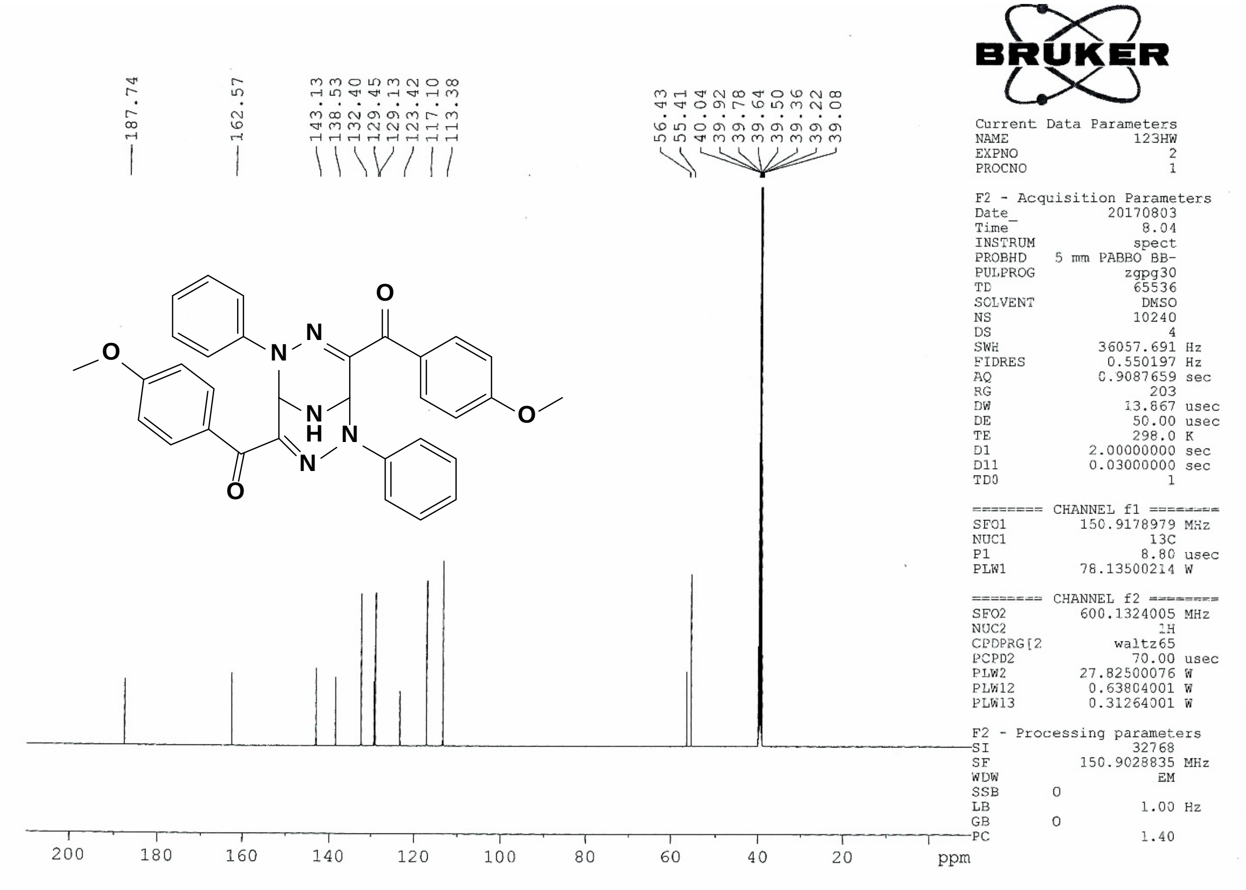

^{1}H NMR spectrum of compound **2k**

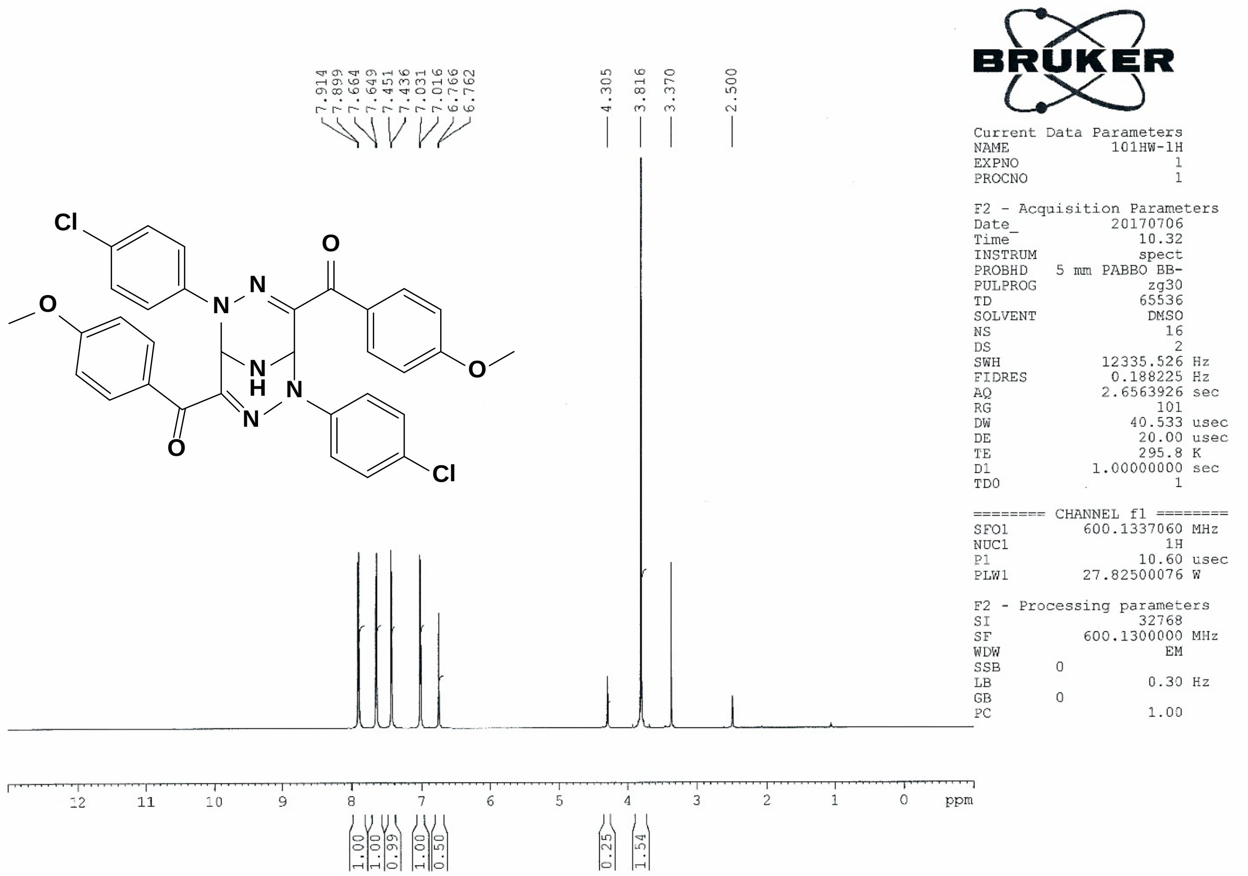

^{13}C NMR spectrum of compound **2k**

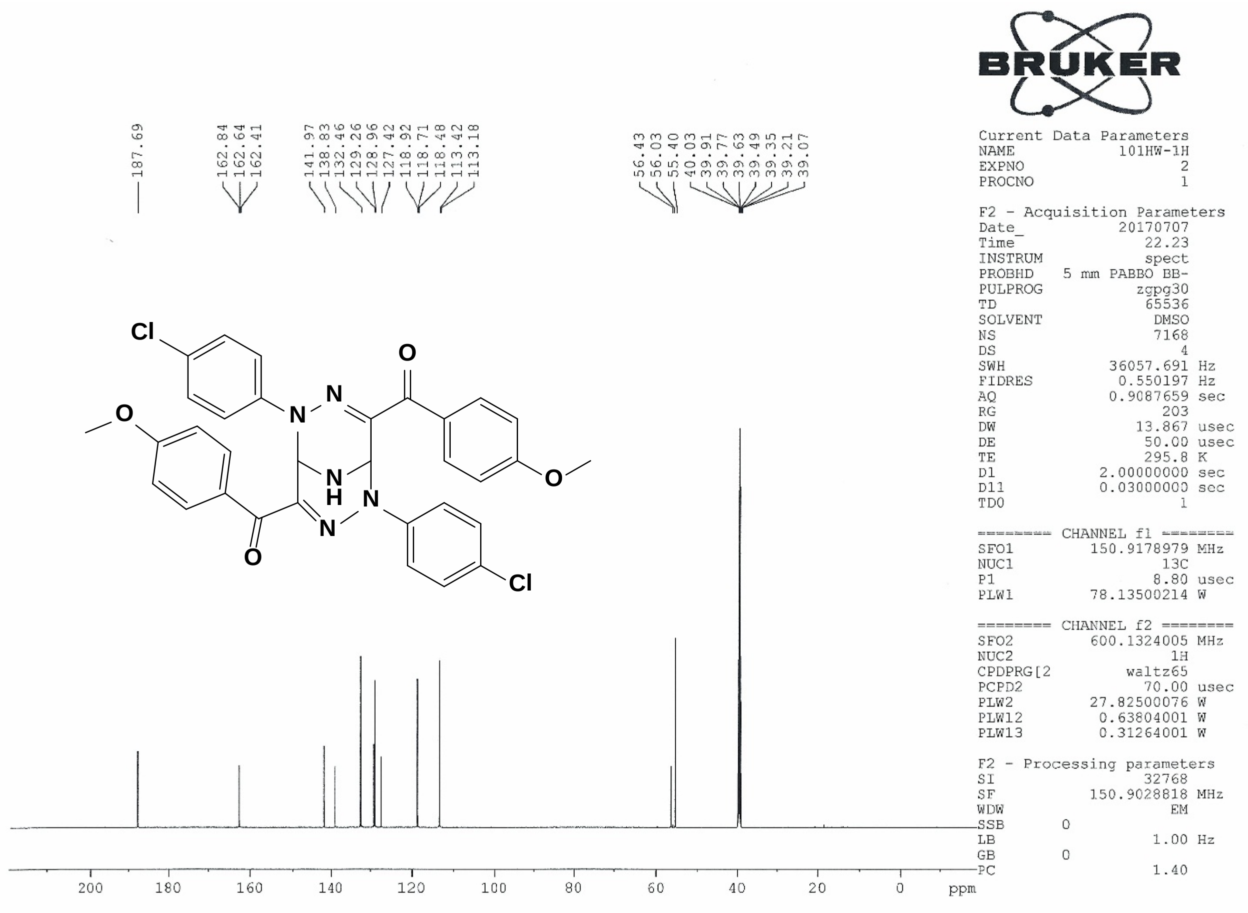

^{1}H NMR spectrum of compound **2l**

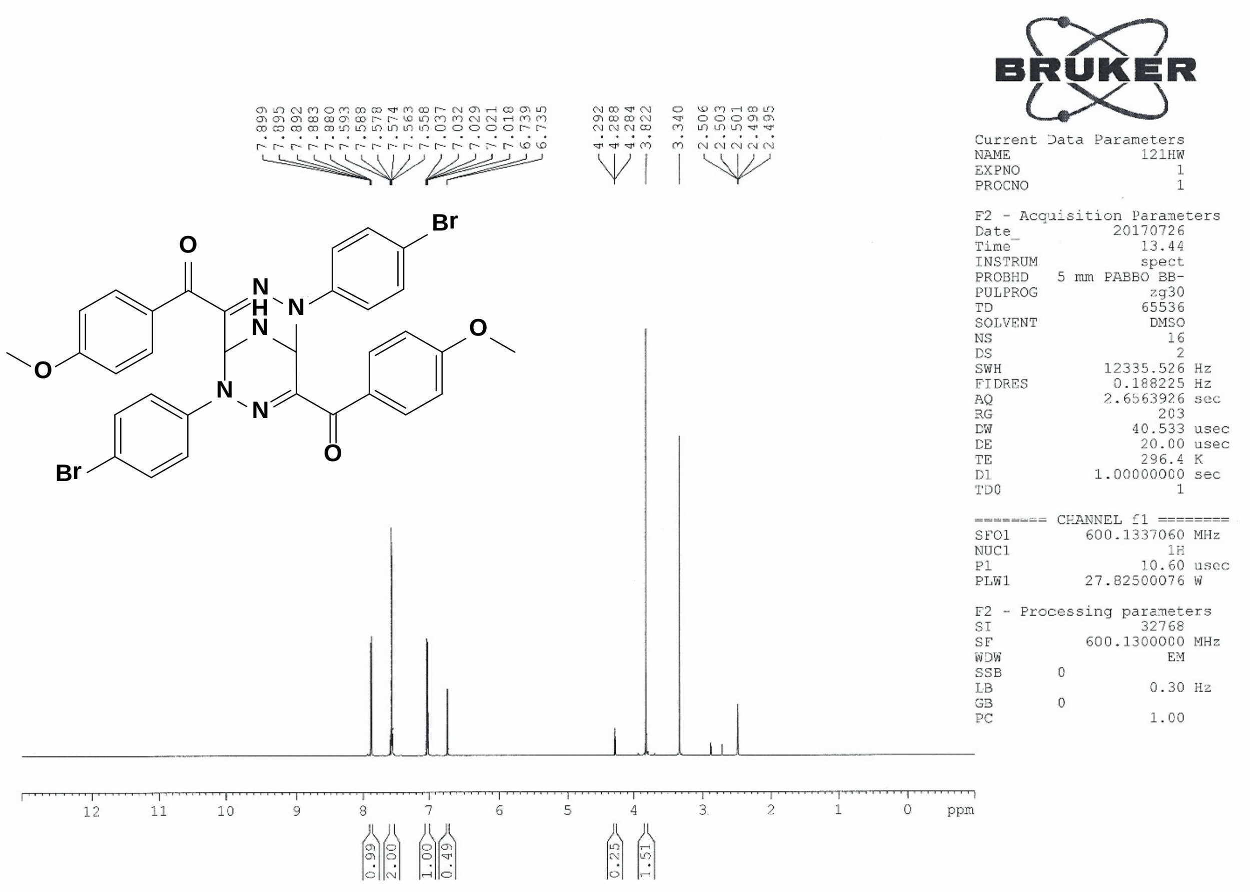

^{13}C NMR spectrum of compound **2l**

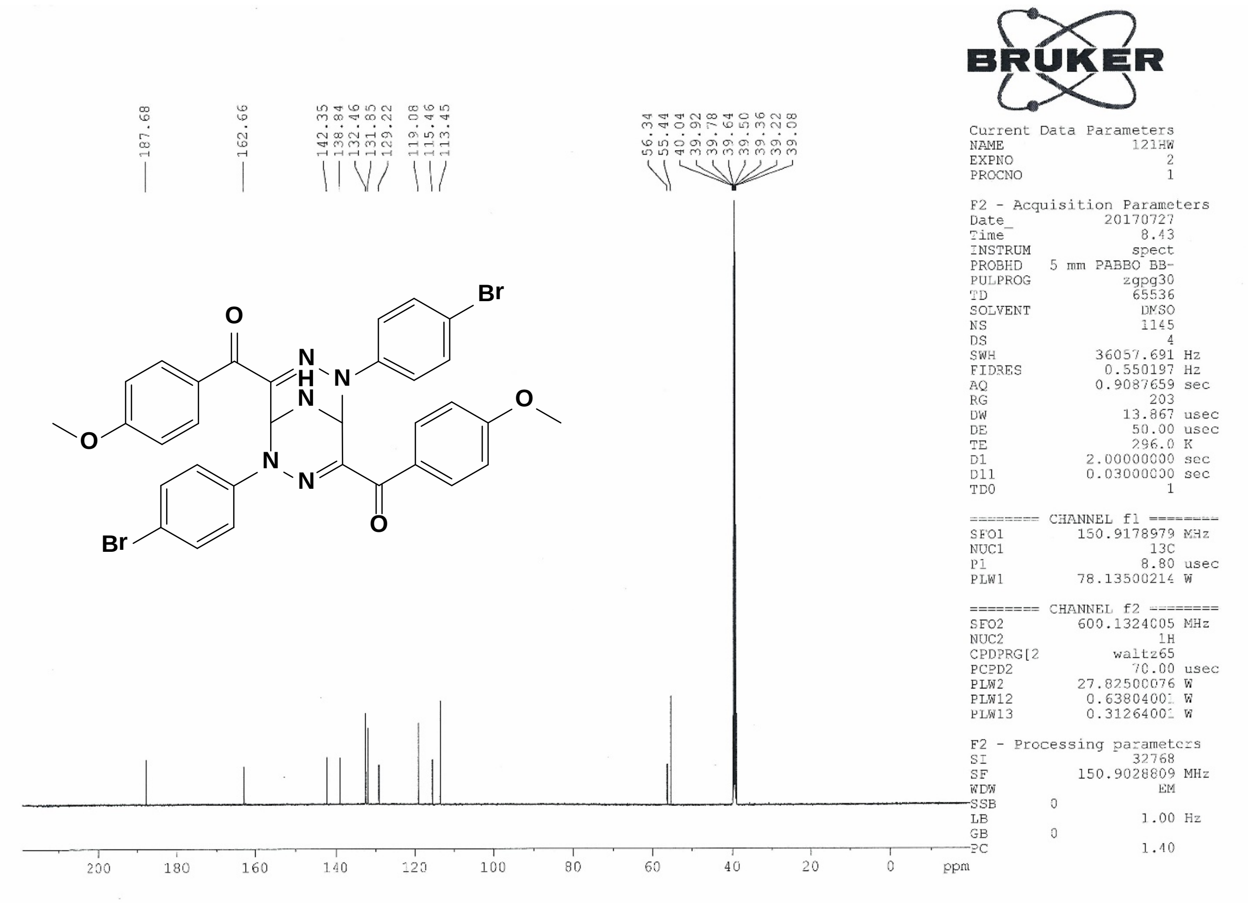

^{1}H NMR spectrum of compound **2m**

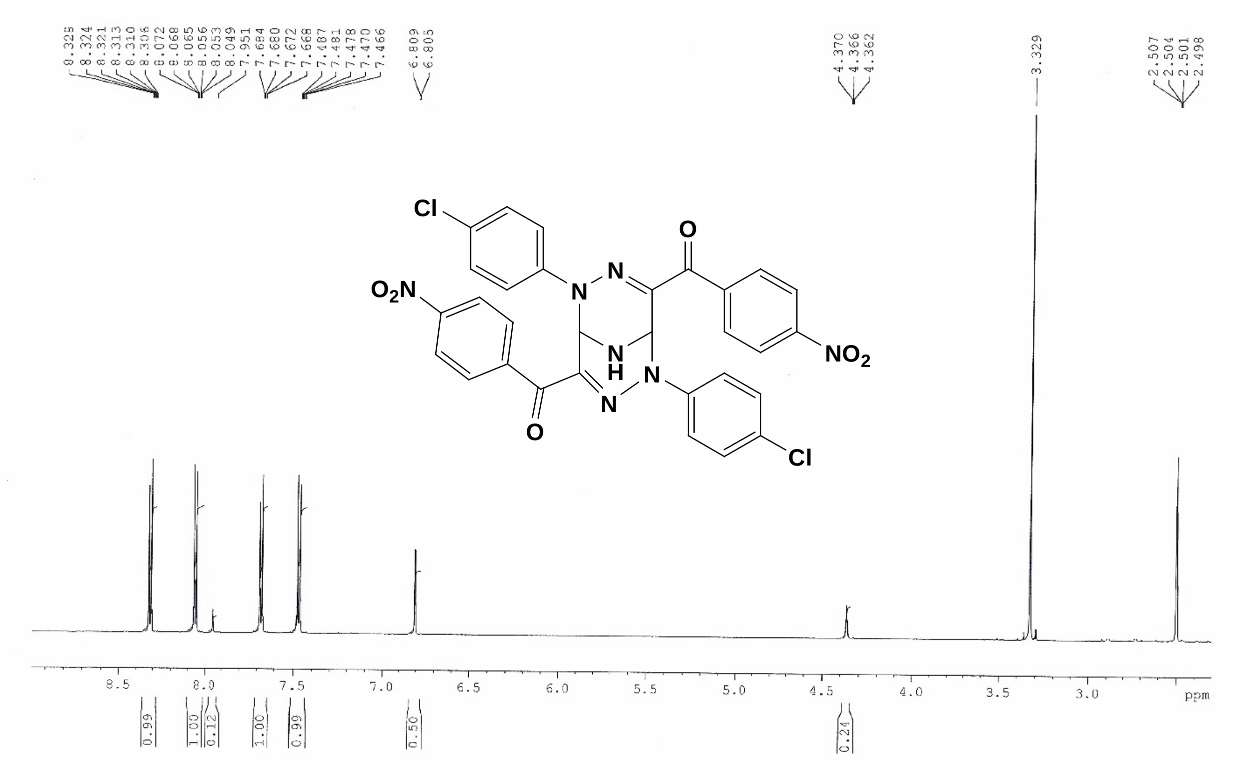

^{13}C NMR spectrum of compound **2m**

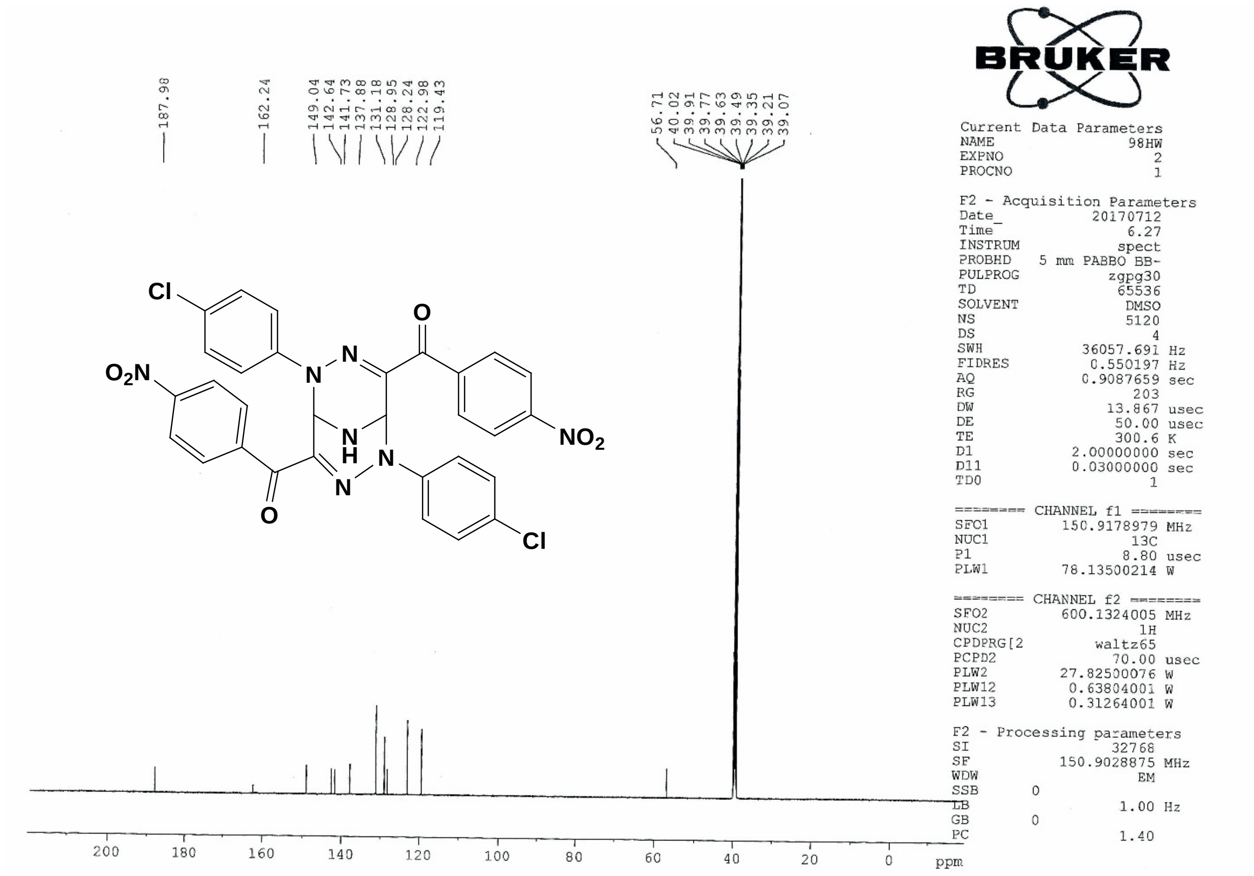

^{1}H NMR spectrum of compound **2n**

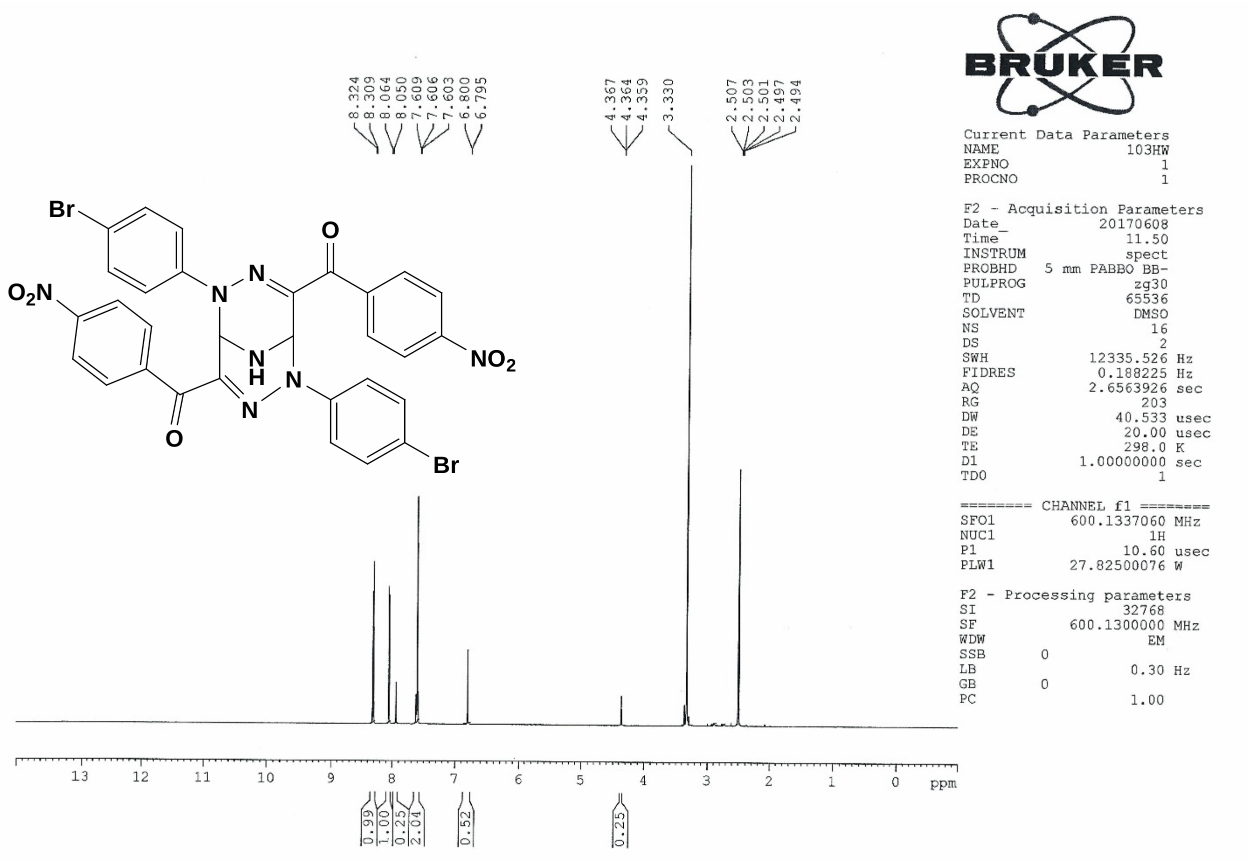

^{13}C NMR spectrum of compound **2n**

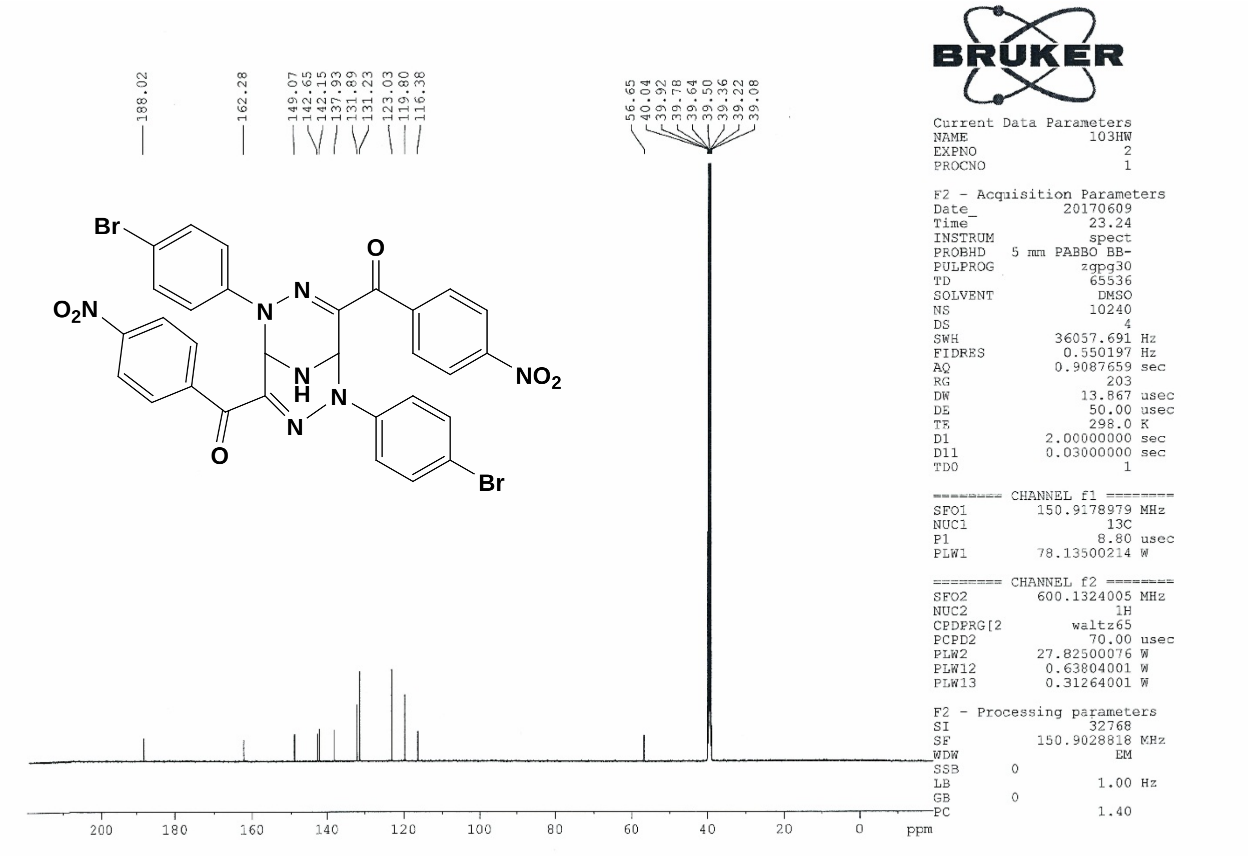

^{1}H NMR spectrum of compound **2o**

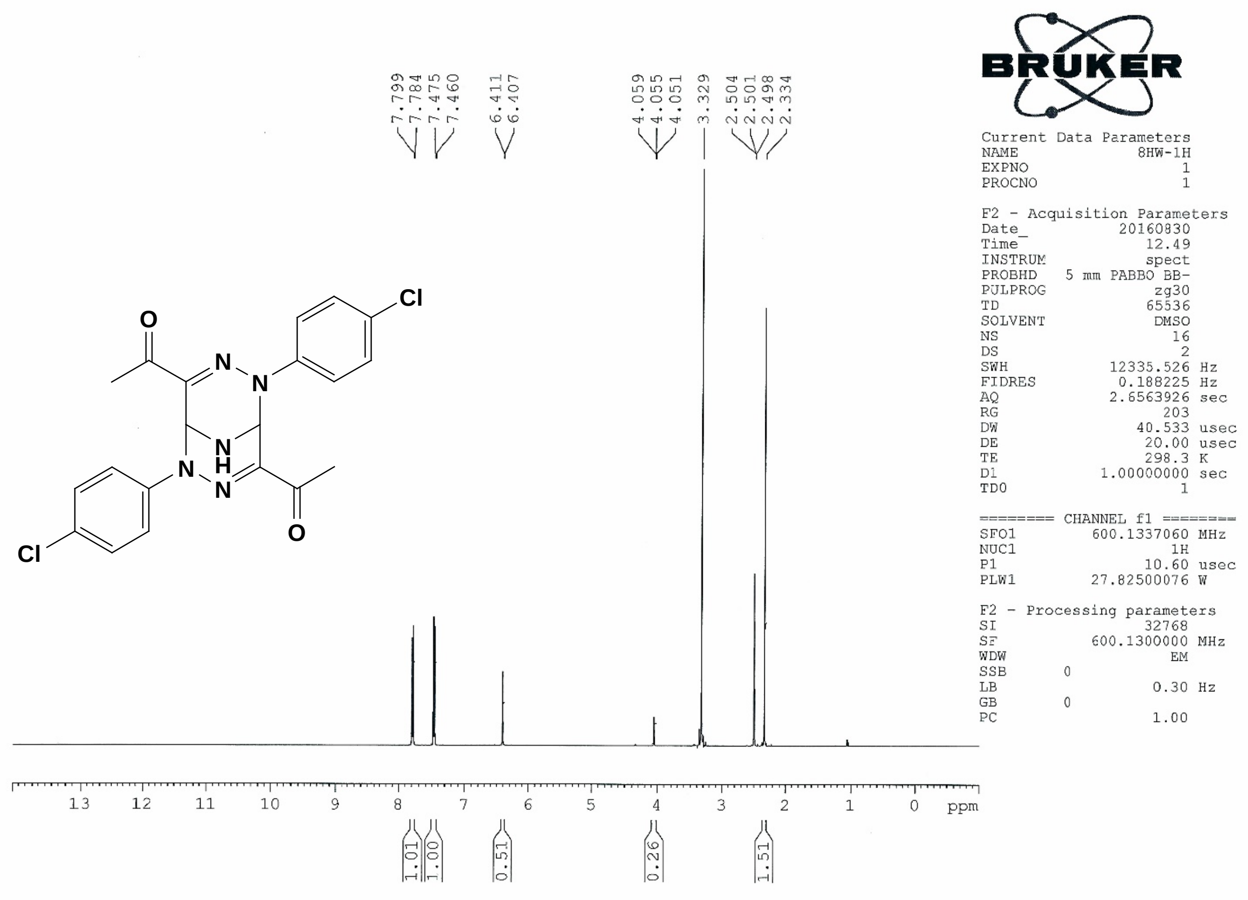

^{13}C NMR spectrum of compound **2o**

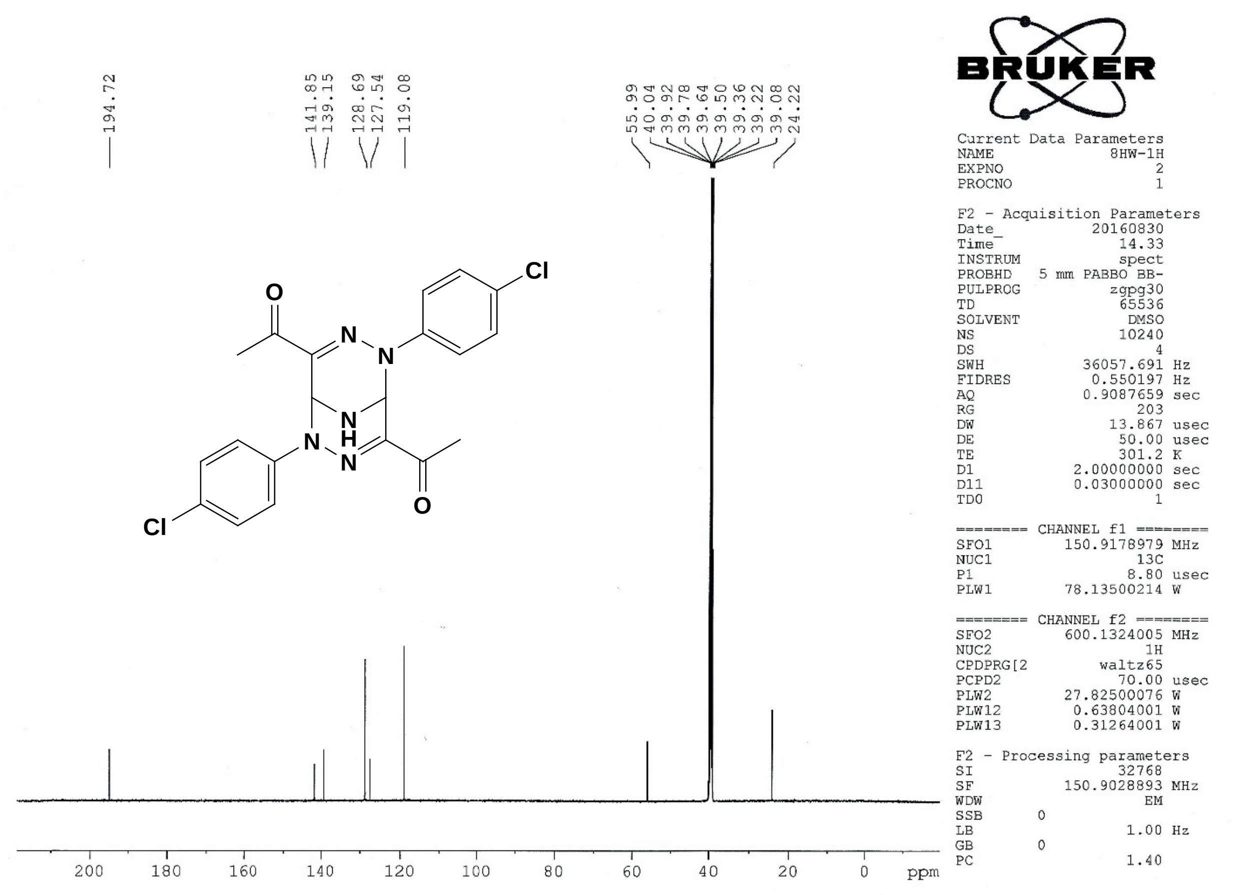

^{1}H NMR spectrum of compound **2p**

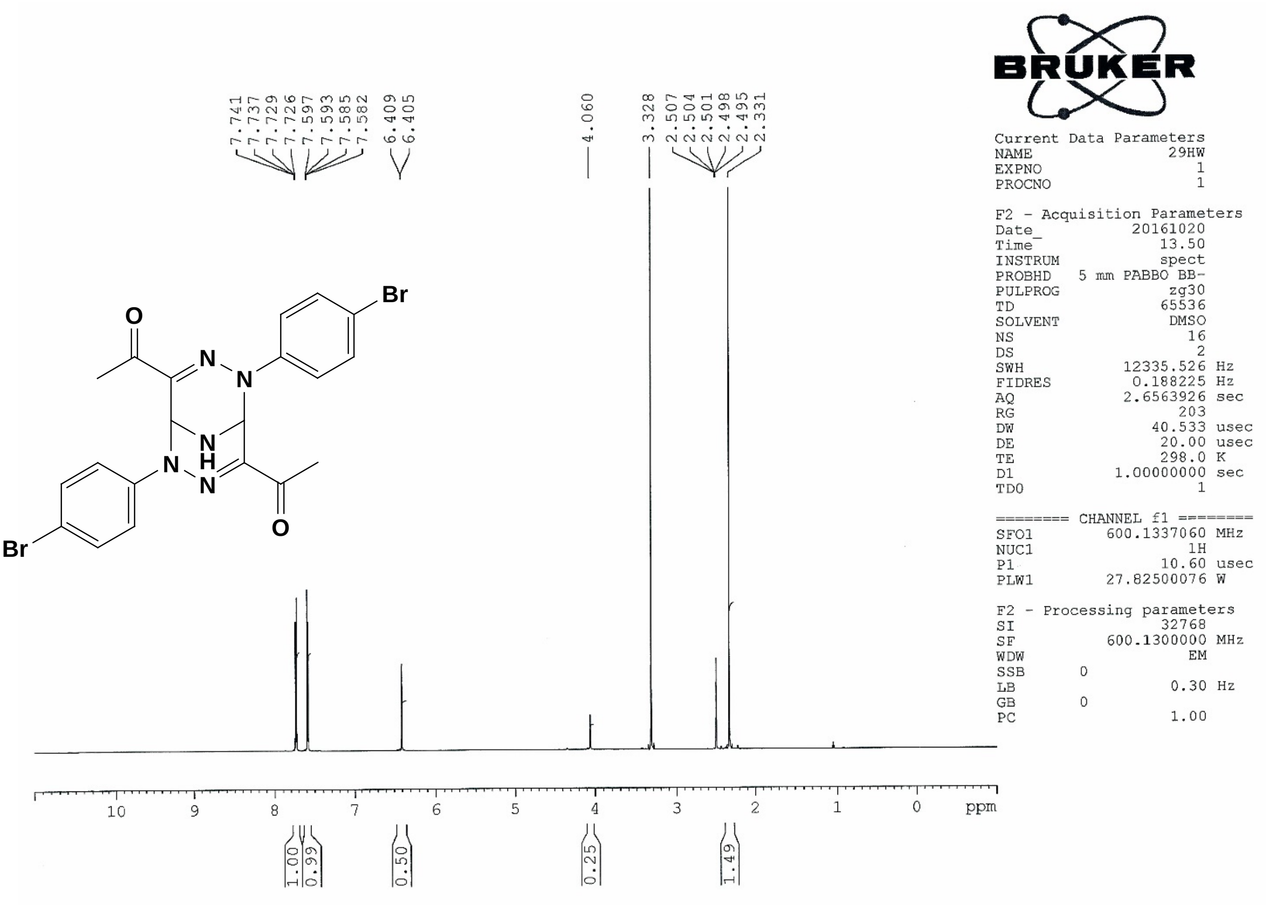

^{13}C NMR spectrum of compound **2p**

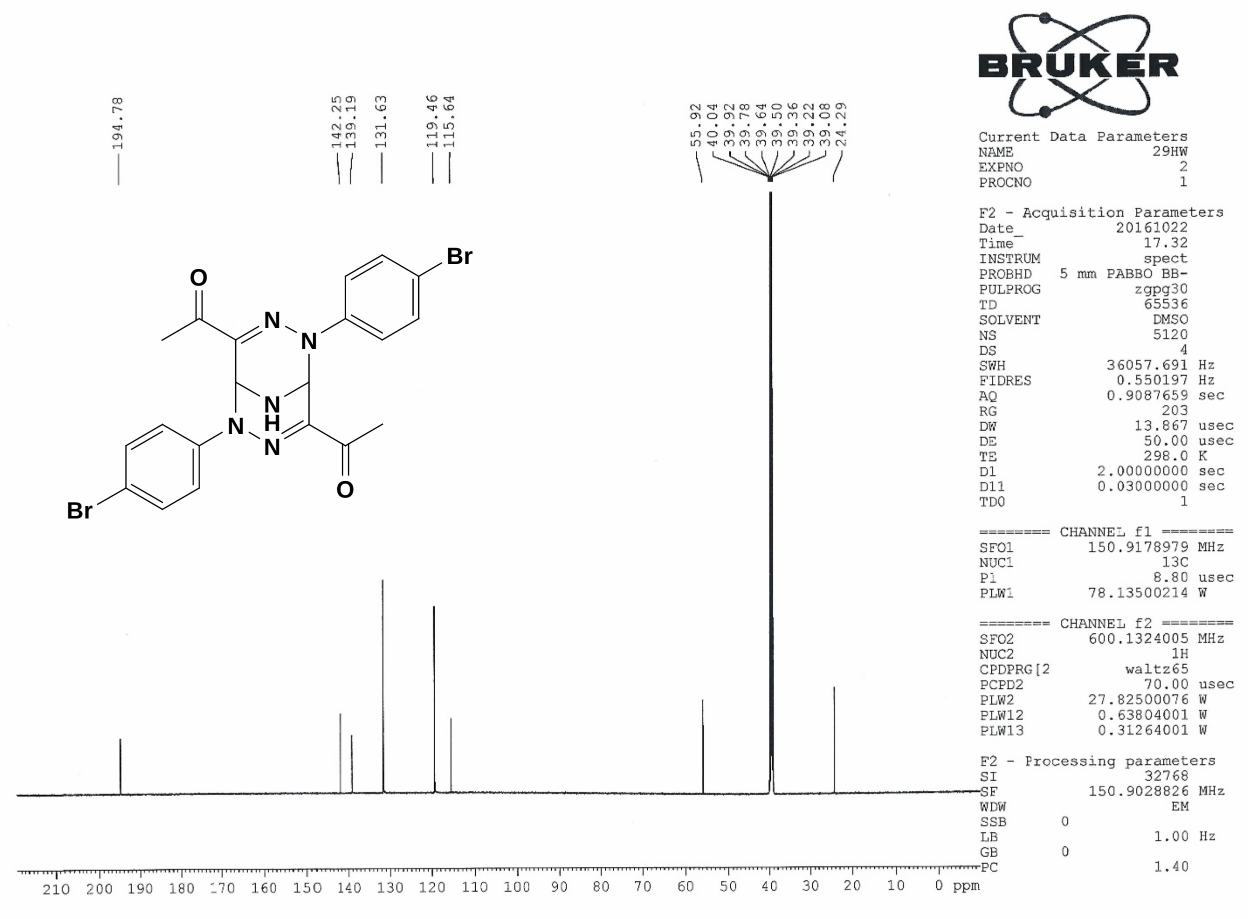

Supplement: Supplementary file 1 [file molecules-24-01110-s001.pdf]
